# Costimulatory Effects of an Immunodominant Parasite Antigen Paradoxically Prevent Induction of Optimal CD8 T Cell Protective Immunity

**DOI:** 10.1371/journal.ppat.1005896

**Published:** 2016-09-19

**Authors:** Christopher S. Eickhoff, Xiuli Zhang, Jose R. Vasconcelos, R. Geoffrey Motz, Nicole L. Sullivan, Kelly O’Shea, Nicola Pozzi, David W. Gohara, Jennifer R. Blase, Enrico Di Cera, Daniel F. Hoft

**Affiliations:** 1 Department of Internal Medicine, Division of Infectious Diseases, Allergy & Immunology, Saint Louis University Medical Center, St. Louis, Missouri, United States of America; 2 Departamento de Biociências, Universidade Federal de São Paulo, Santos, Brazil; 3 Department of Molecular Microbiology and Immunology, Saint Louis University Medical Center, St. Louis, Missouri, United States of America; 4 Department of Biochemistry and Molecular Biology, Saint Louis University Medical Center, St. Louis, Missouri, United States of America; National Institute of Health, UNITED STATES

## Abstract

*Trypanosoma cruzi* infection is controlled but not eliminated by host immunity. The *T*. *cruzi* trans-sialidase (TS) gene superfamily encodes immunodominant protective antigens, but expression of altered peptide ligands by different TS genes has been hypothesized to promote immunoevasion. We molecularly defined TS epitopes to determine their importance for protection versus parasite persistence. Peptide-pulsed dendritic cell vaccination experiments demonstrated that one pair of immunodominant CD4^+^ and CD8^+^ TS peptides alone can induce protective immunity (100% survival post-lethal parasite challenge). TS DNA vaccines have been shown by us (and others) to protect BALB/c mice against *T*. *cruzi* challenge. We generated a new TS vaccine in which the immunodominant TS CD8^+^ epitope MHC anchoring positions were mutated, rendering the mutant TS vaccine incapable of inducing immunity to the immunodominant CD8 epitope. Immunization of mice with wild type (WT) and mutant TS vaccines demonstrated that vaccines encoding enzymatically active protein and the immunodominant CD8^+^ T cell epitope enhance subdominant pathogen-specific CD8^+^ T cell responses. More specifically, CD8^+^ T cells from WT TS DNA vaccinated mice were responsive to 14 predicted CD8^+^ TS epitopes, while T cells from mutant TS DNA vaccinated mice were responsive to just one of these 14 predicted TS epitopes. Molecular and structural biology studies revealed that this novel costimulatory mechanism involves CD45 signaling triggered by enzymatically active TS. This enhancing effect on subdominant T cells negatively regulates protective immunity. Using peptide-pulsed DC vaccination experiments, we have shown that vaccines inducing both immunodominant and subdominant epitope responses were significantly less protective than vaccines inducing only immunodominant-specific responses. These results have important implications for *T*. *cruzi* vaccine development. Of broader significance, we demonstrate that increasing breadth of T cell epitope responses induced by vaccination is not always advantageous for host immunity.

## Author Summary

Chagas disease, driven by persistent infection with the intracellular protozoan parasite *Trypanosoma cruzi*, continues to be a major cause of morbidity and mortality in the Americas. Infections with complex pathogens induce T cell responses to a variety of proteins and epitopes in a specific and sometimes predictable pattern of immune dominance. Extensive research has been conducted with the goal of broadening vaccine-induced T cell responses, for example to better protect against pathogen immune escape due to epitope mutation. We show that *T*. *cruzi* trans-sialidase (TS) induces complex immunodominant and subdominant T cell responses after both *T*. *cruzi* infection and TS vaccination. In order to study the importance of immunodominant and subdominant TS epitopes, we generated TS vaccines designed to abolish T cell responses to the immunodominant TS CD8^+^ T cell epitope. These vaccines indeed abolished CD8^+^ T cell responses to the immunodominant epitope, but interestingly also eliminated T cell responses to many subdominant epitopes. Furthermore, vaccines inducing both immunodominant and subdominant epitope responses were significantly less protective than vaccines inducing only immunodominant-specific responses. Thus, increasing the breadth of T cell epitope recognition may not necessarily enhance protective immunity, and in fact, may be detrimental to the desired goals.

## Introduction

*Trypanosoma cruzi* is an intracellular protozoan parasite and the causative agent of Chagas disease. Approximately 10 million people are infected with *T*. *cruzi*, 25 million are at risk of the infection, and >10,000 die each year from Chagas disease (http://www.who.int). New infections occur through contact with excretions from parasite-infected triatomines (reduviid insects), blood transfusions, and organ donations. To date there are no highly effective vaccines or chemotherapies for the prevention or treatment of *T*. *cruzi* infection and/or disease.

Our group has shown that CD4^+^ Th1 cells (producing IFN-γ and IL-2) are critical both for *T*. *cruzi* mucosal and systemic protection. [1–4] Th1 cells activate macrophage intracellular killing, and help generate and maintain CD8^+^ cytolytic T cells (CTL) that recognize and destroy *T*. *cruzi* infected cells. [5–10] However, transfer of *T*. *cruzi*-specific CD4^+^ Th1 cells alone into SCID mice does not provide protection. Indeed, adoptive transfer of both naïve CD4^+^ and naïve CD8^+^ T cells into SCID mice is absolutely required for reconstitution of immunity against primary *T*. *cruzi* challenges. [2] Furthermore, DNA vaccines must encode both CD4^+^ and CD8^+^ *T*. *cruzi*-specific T cell epitopes to induce protection. [8] In contrast, activated *T*. *cruzi*-specific CD8^+^ effector T cells alone can transfer protection. [10, 11] Overall, these previous results indicate that CD4^+^ Th1 cells provide critical helper effects for the development of protective immunity, while CD8^+^ CTL are required for effector functions protective against *T*. *cruzi* infection.

Trans-sialidase (TS) is an important parasite virulence factor targeted by both antibody and T cell responses during murine and human *T*. *cruzi* infection. [12–15] The TS enzymatic activity includes neuraminidase and sialic acid transfer capacities required for parasite infectivity. [13] The catalytic domain is highly conserved and present in all *T*. *cruzi* parasite strains. In addition, the parasite genome was shown to contain approximately 1400 genes sharing partial homology with the TS enzymatic domain but most lacking enzymatic activity and of unknown function. [16] Many TS superfamily genes have been shown to encode immunodominant T cell epitopes that induce CD8^+^ T cell responses during acute infection that can achieve frequencies as high as 30% of all circulating CD8^+^ T cells. [17] Vaccines encoding TS antigens can protect mice against normally lethal systemic challenges as well as protect against parasite mucosal invasion. [8, 9, 11, 18–22] However, no regimens, including TS vaccinations, have been able to induce sterilizing immunity capable of preventing the establishment of chronic parasite infection. Furthermore, the existence of hundreds of TS partial homologues suggests the evolution of an elaborate immunoevasion strategy providing altered peptide ligands capable of dampening immunity during later stages of *T*. *cruzi* chronic infection. Therefore, detailed molecular studies of TS-specific immune responses are required to determine the true potential for TS vaccine development.

In the current work we explore the roles of a previously described immunodominant TS CD8^+^ T cell epitope (TSKd1; [8, 11, 23, 24]), an immunodominant I-A^d^-restricted CD4^+^ epitope recently identified using overlapping peptide arrays shown to elicit the most potent CD4^+^ T cell responses of any peptide tested after TS vaccination (p7; manuscript in preparation), as well as subdominant CD8^+^ T cell epitopes described in this manuscript which were identified using MHC binding algorithms (termed “subdominant” TS CD8^+^ T cell epitopes). We show that the single pair of immunodominant CD4^+^ and CD8^+^ T cell epitopes within the consensus TS enzymatic domain are minimally sufficient for induction of protective *T*. *cruzi* immunity. We also demonstrate that in addition to responses directed against the immunodominant CD8^+^ epitope, TS vaccines induce a complex molecular set of TS epitope-specific responses. These subdominant epitopes can inhibit protective immunity, and the mechanism for these effects involves enzymatically active TS-induced CD45-mediated costimulation. These results have important implications for both *T*. *cruzi* vaccine development and more broadly for the development of epitope-based vaccines.

## Results

### The TS derived p7 CD4 and TSKd1 CD8 T cell epitopes alone can induce potently protective *T*. *cruzi* immunity

**Fig 1:**
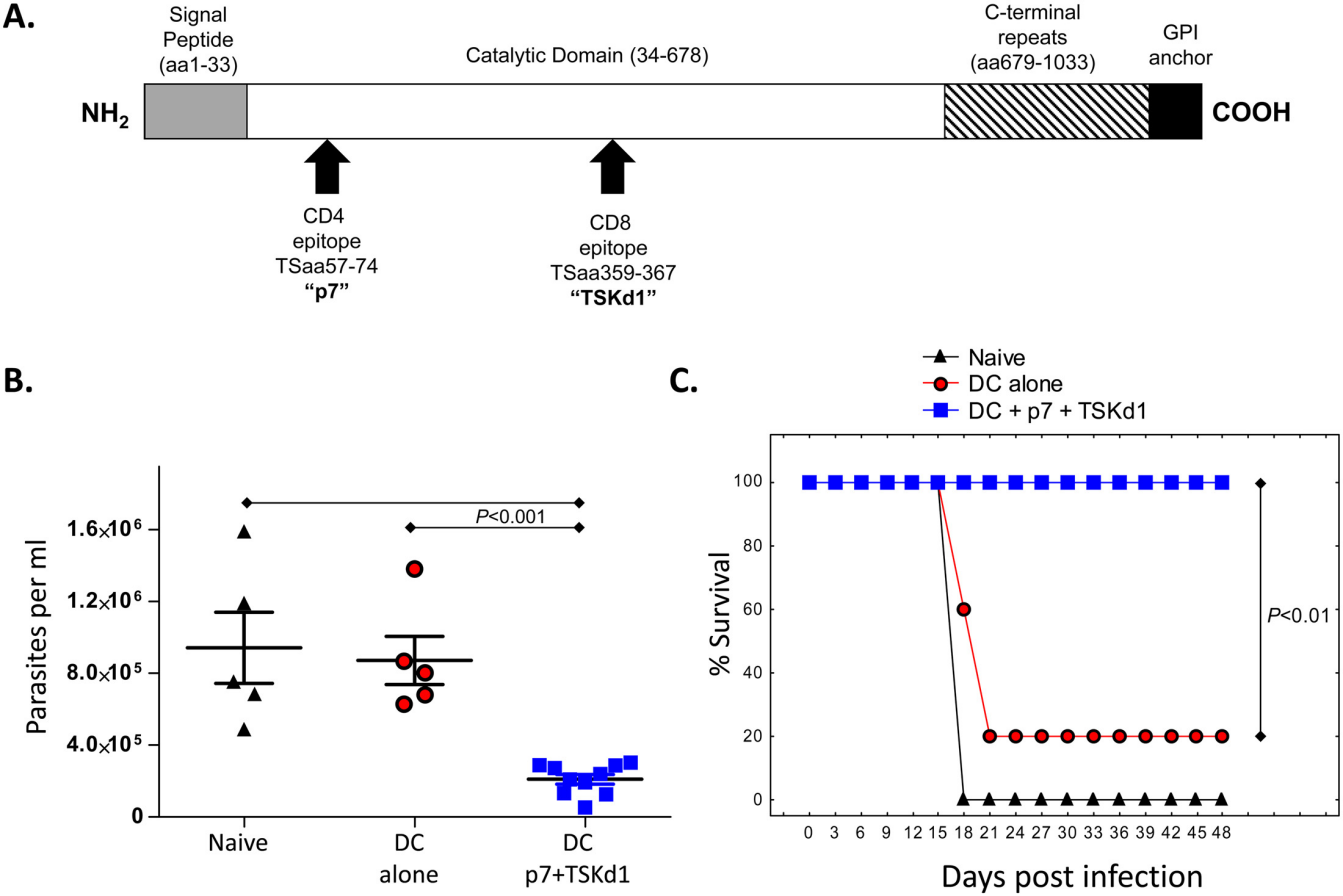
CD4^+^ TSaa57-74 (p7)/IA^d^- and CD8^+^ TSaa359-367 (TSKd1)/K^d^-specific T cell responses are minimally sufficient for induction of protective *T*. *cruzi* immunity. Panel A shows a schematic of the TS consensus protein (all 12–15 active TS subfamily members have at least 90% homology within their catalytic domains), and immunodominant TS CD4 and CD8 epitopes. In panels B and C, BALB/c mice were vaccinated with dendritic cells (DC) pulsed (or not) with this pair of CD4 and CD8 epitopes and later challenged with *T*. *cruzi*. BALB/c CD11c^+^ splenic DC were purified from BALB/c mice 2 weeks after injection of 5x10^6^ B16-Flt3L-producing cells and 1 day after i.v. injection of 1μg LPS. 1x10^6^ DC pulsed (or left unpulsed) with 50 μg/ml of the indicated peptides were injected i.v. into groups of naïve BALB/c mice 3 times, 2 weeks apart. Mice were challenged 1 month later with *T*. *cruzi* (N = 5 in each of the two control groups and N = 10 in DC+TS peptide group). Both parasitemia (B; 2 weeks post-infection) and mortality (C) were significantly reduced in mice given DC pulsed with both TSaa57-74 (p7) and TSaa359-367 (TSKd1) [*p<0.001 by Mann-Whitney U test (B) and *p<0.01 by 2-tailed Fisher exact and Log-Rank [Mantel-Cox] tests(C)]. Survival results are representative of 3 independent experiments.

It has been demonstrated previously that both CD4^+^ and CD8^+^ T cells are required for induction of immunity protective against *T*. *cruzi* challenge.[2, 8] A TS immunodominant H2-K^d^-restricted CD8^+^ T cell epitope (TSKd1; IYNVGQVSI) [8, 23, 24] and newly identified I-A^d^-restricted CD4^+^ T cell epitope (p7; KVTERVVHSFRLPALVNV) were utilized to further explore the importance of both CD4^+^ and CD8^+^ epitopes for TS-specific protective immunity (Fig 1A). We conducted immunization and challenge experiments, vaccinating mice with dendritic cells (DC) pulsed only with the TS peptides TSKd1 and p7. Groups of 5–10 BALB/c mice were vaccinated intravenously with 1x10^6^ peptide-pulsed DC, at least twice at 1–2 week intervals, and then challenged systemically with highly virulent *T*. *cruzi* parasites four weeks later (Fig 1B and 1C). Control mice were either unvaccinated or vaccinated with unpulsed DC. Mice vaccinated with DC pulsed with TSKd1 and p7 displayed significantly reduced parasitemia 2 weeks post-infection compared to both naïve controls and non-peptide pulsed DC vaccinated mice (Fig 1B; p<0.001 by Mann-Whitney U test). In addition all mice vaccinated with the TSKd1 and p7 peptide-pulsed DC were protected and survived, whereas both control groups suffered high mortality post-challenge (Fig 1C; p<0.01 comparing DC+p7+TSKd1-vaccinated mice with both negative control groups by 2-tailed Fisher exact and Log Rank tests). These data confirm that TSKd1-specific CD8^+^ T cells and p7-specific CD4^+^ T cells are minimally sufficient for TS vaccine-induced protective *T*. *cruzi* immunity.

### TSKd1 (TSaa359-367) is an immunodominant protective CD8^+^ T cell epitope

**Fig 2:**
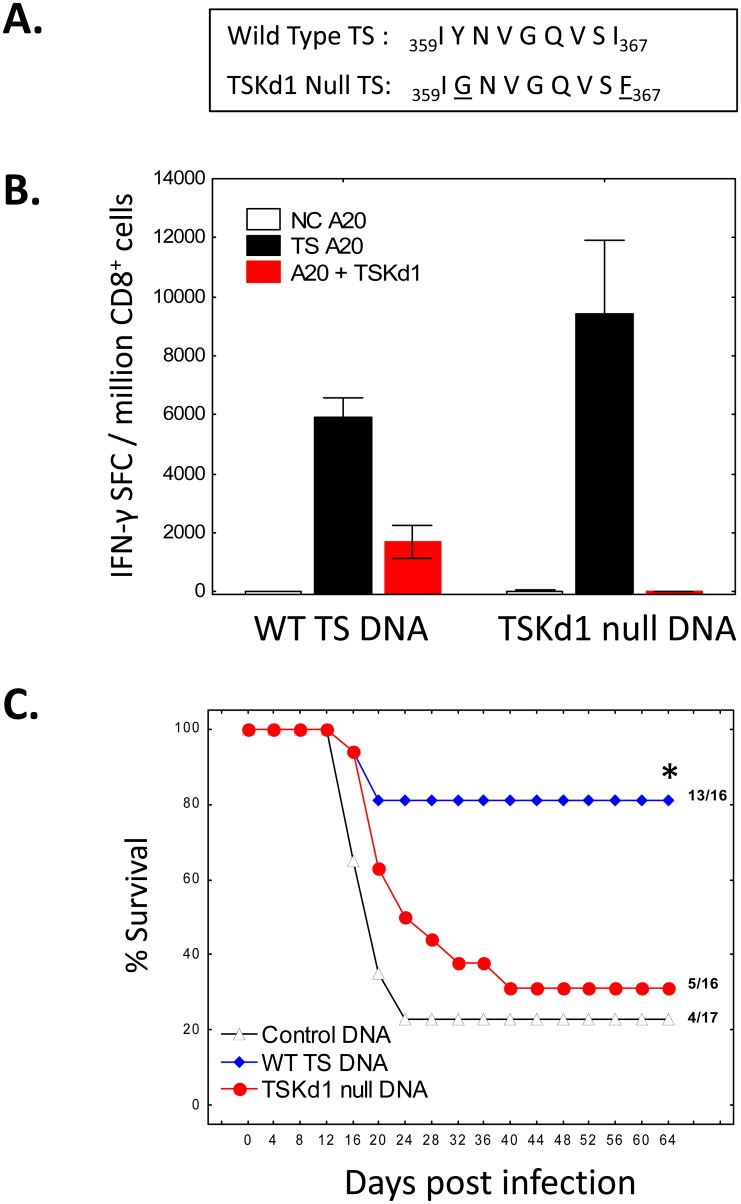
TSKd1-specific CD8^+^ T cell responses provide immunodominant protective activity. To understand whether or not the TSaa359-367 peptide (TSKd1) is immunodominant and absolutely required for induction of protective *T*. *cruzi* immunity, a site-directed mutagenesis strategy was utilized (A) to completely remove predicted binding of TSKd1 to H2-K^d^. Groups of BALB/c mice were vaccinated twice, 2 weeks apart with negative control DNA, the WT TS DNA (WT TS DNA) or the new TSKd1 null TS DNA (TSKd1 null DNA). One month after the final vaccination, pooled splenic CD8^+^ T cells were obtained from representative vaccinated mice and stimulated with control APC (NC A20), APC transfected with TS aa1-678 (TS A20), or APC pulsed with TSKd1 (A20 +TSKd1) in overnight IFN-γ ELISPOT assays (B). Other groups of vaccinated mice (N = 16–17 each) were challenged with 5,000 *T*. *cruzi* BFT s.c. 4 weeks following the final immunization and survival monitored (C). *, p<0.05 compared with NC DNA and CD8 Null TS DNA groups by 2-tailed Fisher exact tests and Log-Rank [Mantel-Cox] analyses. Results are representative of at least 3 experiments.

The TSKd1 epitope has been shown to induce protective CD8^+^ T cell responses [Fig 1 and [8]]. To determine whether the TSKd1 epitope is the only TS CD8^+^ epitope involved in protective immunity, we performed site-directed mutagenesis of the TS DNA vaccine (comprised of TS catalytic domain amino acids 1–678). We altered the TSKd1 2^nd^ position tyrosine to a glycine and 9^th^ position isoleucine to a phenylalanine, creating a new “TSKd1 null” TS DNA vaccine (Fig 2A). The TSKd1 null vaccine is identical to our wild type (WT) TS DNA vaccine except for these 2 amino acid changes. These changes were predicted to completely abolish binding of the TSKd1 epitope to H-2K^d^, and indeed the TSKd1 null vaccine failed to induce any detectable T cell responses directed against the TSKd1 epitope (Fig 2B). However, to our surprise BALB/c mice vaccinated with the WT and TSKd1 null vaccines developed similar total and CD8^+^ T cell responses reactive with the entire WT TS consensus catalytic domain (S1 and S2 Figs and Fig 2B). Despite inducing similar overall TS-specific total CD8^+^ T cell responses, only the WT TS vaccine was protective against virulent systemic *T*. *cruzi* challenges (Fig 2C). After challenge, 13 of 16 (81%) WT TS-vaccinated, 5 of 16 (31%) TSKd1 null-vaccinated and 4 of 17 (24%) negative control vector-vaccinated mice survived ≥2 months post-challenge (p<0.05 comparing WT TS-vaccinated with the other groups by 2-tailed Fisher exact tests). These results clearly demonstrate that the TSKd1 CD8 epitope is an immunodominant TS epitope critical for the induction of protective TS immunity in the murine H-2^d^ background.

### Unexpected complexity of TS-specific CD8^+^ T cell responses

**Fig 3:**
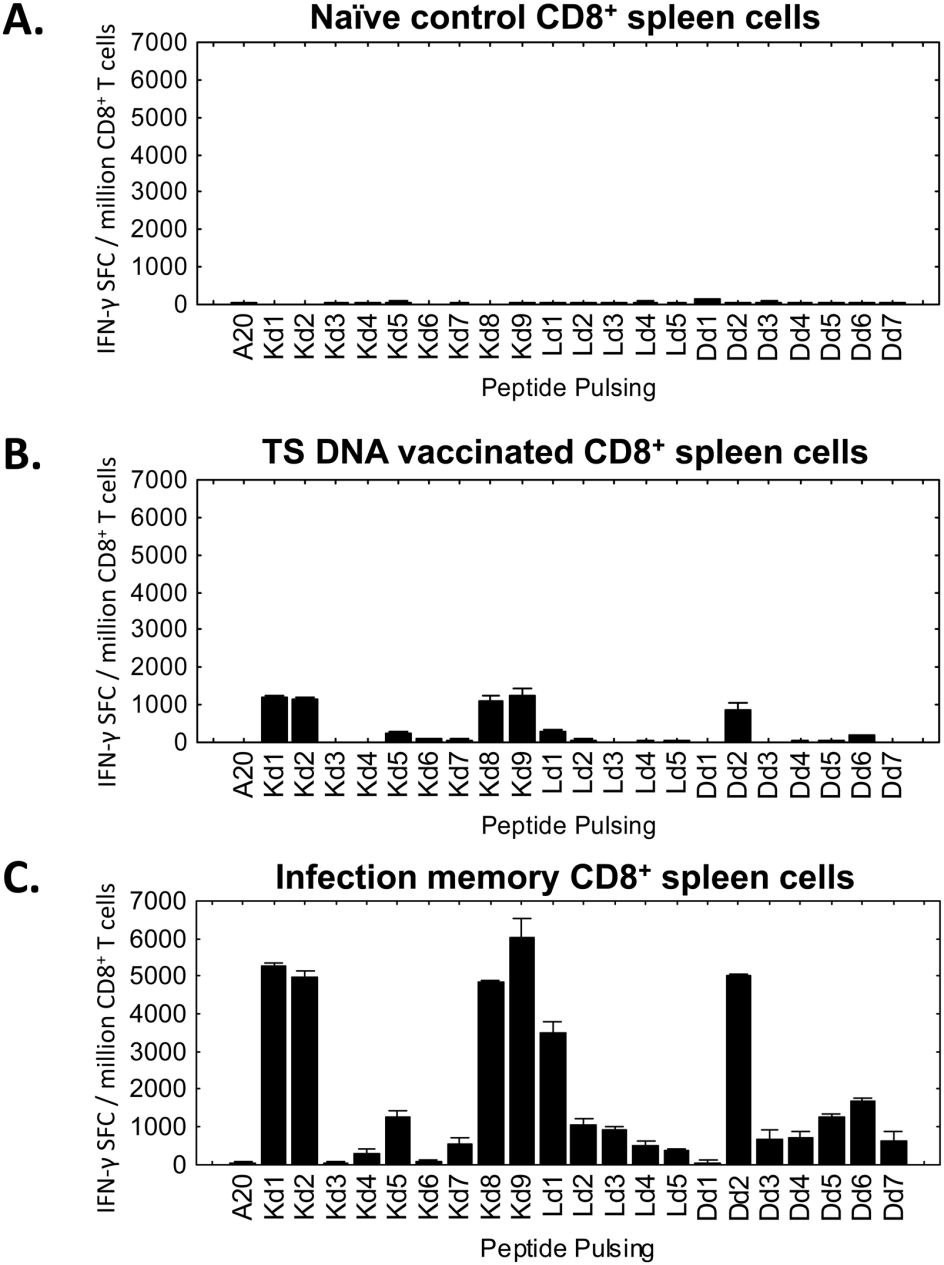
Identification of *trans*-sialidase MHC class I-restricted T cell epitope(s). TSaa359-367 (TSKd1) has been observed to be an immunodominant epitope during both TS vaccination and *T*. *cruzi* infection. We utilized a consensus immunoinformatic approach using several MHC prediction tools to identify other TS sequences predicted to bind BALB/c MHC (H2-K^d^, H2-D^d^, and H2-L^d^, shown in S1 Table). APC (A20) were pulsed with these synthetic peptides and used to stimulate purified splenic CD8^+^ T cells from naïve (A), WT TS DNA-vaccinated (B), and *T*. *cruzi* “Infection Memory” mice (C) in overnight IFN-γ ELISPOT assays. Infection Memory (C) was induced by multiple, virulent Tulahuen strain *T*. *cruzi* challenges, resulting in mice with potently protective T cell immunity directed against a variety of parasite antigens and epitopes. Nearly all of the predicted peptides elicited memory T cell IFN-γ responses in mice previously infected with *T*. *cruzi* and mice vaccinated with WT TS DNA. Results are representative of 3 independent experiments.

Because similar total CD8^+^ T cell responses were induced by the protective WT and non-protective TSKd1 null TS vaccines, we next investigated the molecular detail of CD8 epitope responses induced by these vaccines. We hypothesized that comparing the responses induced by these vaccines would identify both epitopes involved in protective immunity and the epitopes important for *T*. *cruzi* immunoevasion. We used 5 different publicly available, web-based T cell epitope prediction programs to identify TS sequences for testing (S1 Table). We identified 9 peptides predicted to be presented by H-2K^d^, 7 peptides predicted to be presented by H-2D^d^ and 5 peptides predicted to be presented by H-2L^d^ MHC alleles. We labeled these peptides as TSKd1-9, TSDd1-7, and TSLd1-5, based on their average relative rankings across all 5 T cell epitope prediction programs. TSKd1 was previously validated as a bonafide TS-specific CD8^+^ T cell stimulating epitope important for protective *T*. *cruzi* immunity [8, 9], and was shown to be an immunodominant epitope during natural parasite infections. [24, 25] TSKd1-specific CD8^+^ T cell frequencies have achieved levels as high as 40% of the total CD8^+^ T cells after TS vaccination and *T*. *cruzi* infection in BALB/c mice. [11] None of the other epitopes predicted by our immunoinformatic strategy had been shown to be immunogenic previously in either vaccination or infection studies. Fig 3 demonstrates that TSKd1 and many of the other predicted epitopes induce memory CD8^+^ T cell responses in both mice with WT TS DNA vaccine-induced T cell memory (Fig 3B) and mice with *T*. *cruzi* infection-induced T cell memory (Fig 3C). Therefore, these results demonstrate that the H-2^d^-restricted CD8^+^ T cell responses induced by TS vaccination and *T*. *cruzi* infection are molecularly complex.

### WT TS DNA vaccine uniquely induces complex immunodominant and subdominant TS-specific CD8^+^ T cell responses

**Fig 4:**
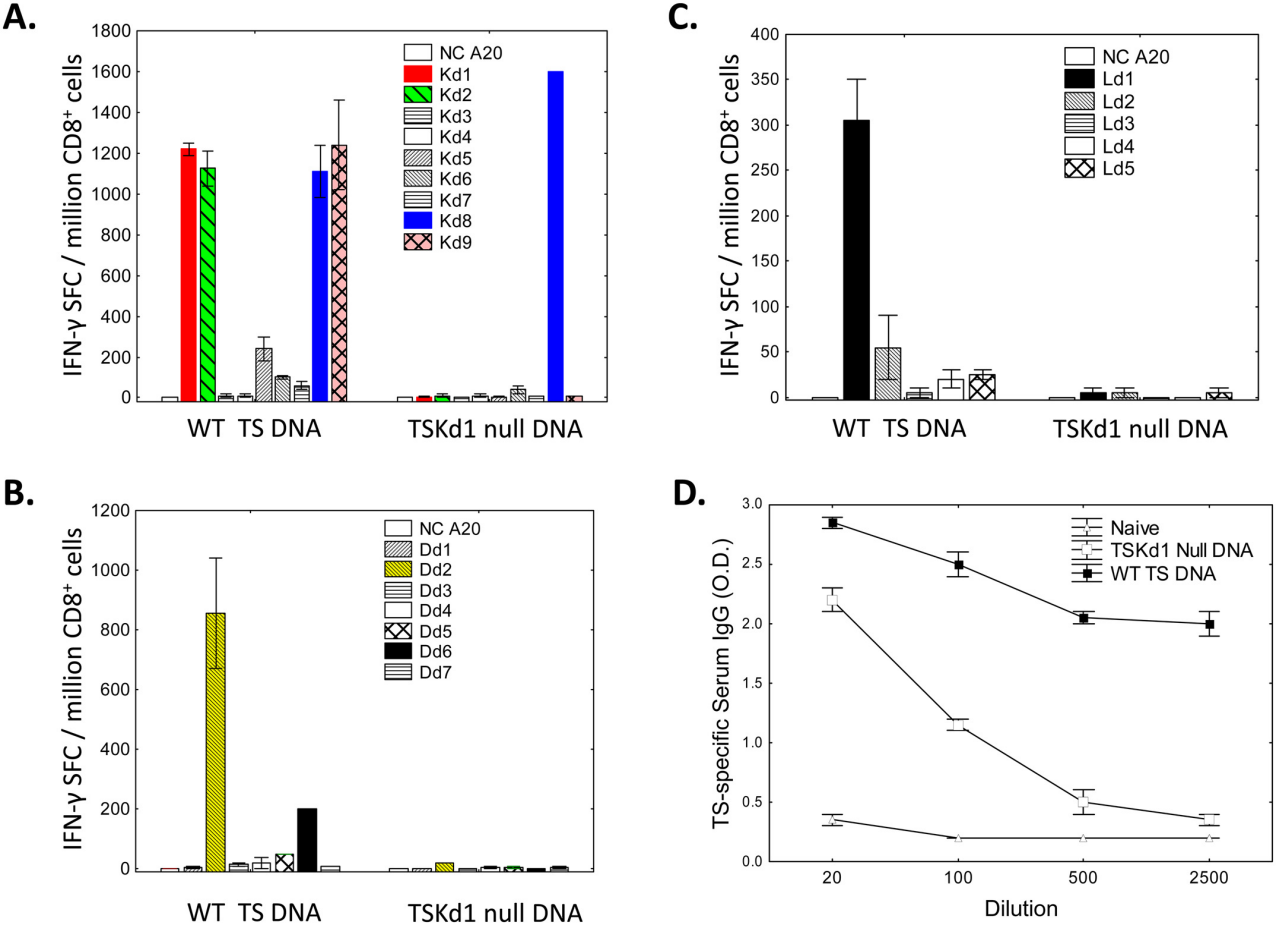
The WT DNA vaccine induces CD8^+^ T cell responses directed against both immunodominant and subdominant T cell epitopes. BALB/c mice were vaccinated i.m. twice, two weeks apart with 100μg of WT TS DNA or TSKd1 null TS DNA. Four weeks later, CD8^+^ splenic T cells from these vaccinated mice were stimulated overnight in IFN-γ ELISPOT assays with APC (A20 cells) pulsed with TS peptides predicted to bind BALB/c MHC [H2-K^d^ (A), H2- D^d^ (B) and H2-L^d^(C). Results are representative of 3 experiments. As expected, vaccination of mice with WT but not TSKd1 null TS DNA constructs elicited T cell responses to TSKd1. CD8^+^ T cells from WT TS DNA vaccinated mice also responded to TS peptides Kd2, Kd5, Kd6, Kd7, Kd8, Kd9, Dd2, Dd6, Ld1 and Ld2, while cells from TSKd1 null vaccinate mice responded to only TS Kd8. Mutation of the 2 binding residues of TSKd1 to H2-Kd1 resulted in a marked immunofocusing of CD8^+^ T cell responses. Panel D further shows that WT TS DNA vaccination induced higher levels of TS-specific antibody, compared with TSKd1 null DNA vaccination.

The above results demonstrated that the TSKd1 CD8 epitope is an immunodominant protective TS epitope in BALB/c mice, but we also wanted to determine the importance of the other TS encoded K^d^-, D^d^- and L^d^-restricted epitopes (S1 Table and Fig 3) for optimal TS-specific protective immunity. To this end we first conducted detailed epitope specificity studies in mice vaccinated with our WT TS and TSKd1 null TS vaccines. Fig 4 presents the surprising results detected in these experiments. Fig 4A demonstrates that the WT TS vaccine induced T cell responses directed against 7 of 9 of the predicted K^d^-restricted TS epitopes as seen before (Fig 3). However, the TSKd1 null vaccine only induced T cell responses directed against TSKd8, and not responses directed against the other 6 K^d^-restricted CD8 epitopes induced by WT TS vaccination. The WT TS DNA vaccine also induced a more complex pattern of responses to TS-specific subdominant epitopes predicted to be presented by both D^d^ and L^d^ MHC class I molecules (Fig 4B and 4C). Furthermore, the TS vaccine induced significantly higher titers of TS-specific antibodies than induced by the TSKd1 null vaccine (Fig 4D). Overall, these results indicate that instead of having the usual negative impact on subdominant T cell responses, the induction of immunodominant TSKd1 epitope-specific responses paradoxically was associated with a more complex development of CD8^+^ T cells specific for subdominant TS epitopes restricted by all 3 MHC class I alleles expressed in BALB/c mice. In addition, the WT TS vaccine induced a stronger TS-specific antibody response.

### Both the WT TS and TSKd1 null TS vaccines produce similar amounts of TS protein with similar structures

Trivial explanations for the reduced immune responses induced by the TSKd1 null vaccine shown above include the possibilities that the TSKd1 null vaccine produced reduced levels of TS antigen in vivo or a distinct structure processed differently by antigen presenting cells leading to destruction of the subdominant T cell epitopes and/or conformational B cell epitopes. However, we have already shown that similar overall T cell responses were induced by both WT and TSKd1 null vaccines (Fig 2B and S1 and S2 Figs), suggesting that both vaccines produced similar amounts of antigenic protein and presented similar levels of TS encoded CD8^+^ T cell epitopes. To confirm that the TSKd1 null vaccine did not produce reduced levels of protein, or a protein with a distinct structure processed differently, we further compared the 2 vaccine constructs using both in vitro physical studies with proteins expressed by transient transfection and in silico structural predictions. Transient transfections of 293T cells further confirmed that both the WT and TSKd1 null vaccine constructs produced similar overall levels of TS protein with identical B cell epitopes (S3 Fig). Lysates of these transiently transfected cells were used for immunoprecipitations with antibodies collected from the serum of mice vaccinated with either the WT or TSKd1 null vaccines. In S3 Fig, it is clearly shown that similar amounts of TS protein were immunoprecipitated from lysates of 293T cells transfected with either the WT or TSKd1 null vaccine using serum from mice vaccinated with the WT vaccine. Similarly, both the WT and TSKd1 derived proteins were immunoprecipitated with serum from mice vaccinated with the TSKd1 null vaccine. Next, the in silico predicted structures of WT and TSKd1 null TS proteins were compared (S3 Fig). Although mutation of TSKd1 resulted in a predicted shift of the artificial signal peptide (amino acids 1–33), the overall predicted tertiary structures of WT TS and TSKd1 null TS sequences were very similar.

### Tolerization experiments also demonstrate a paradoxical association between TS-specific immunodominant and subdominant CD8^+^ T cell responses

**Fig 5:**
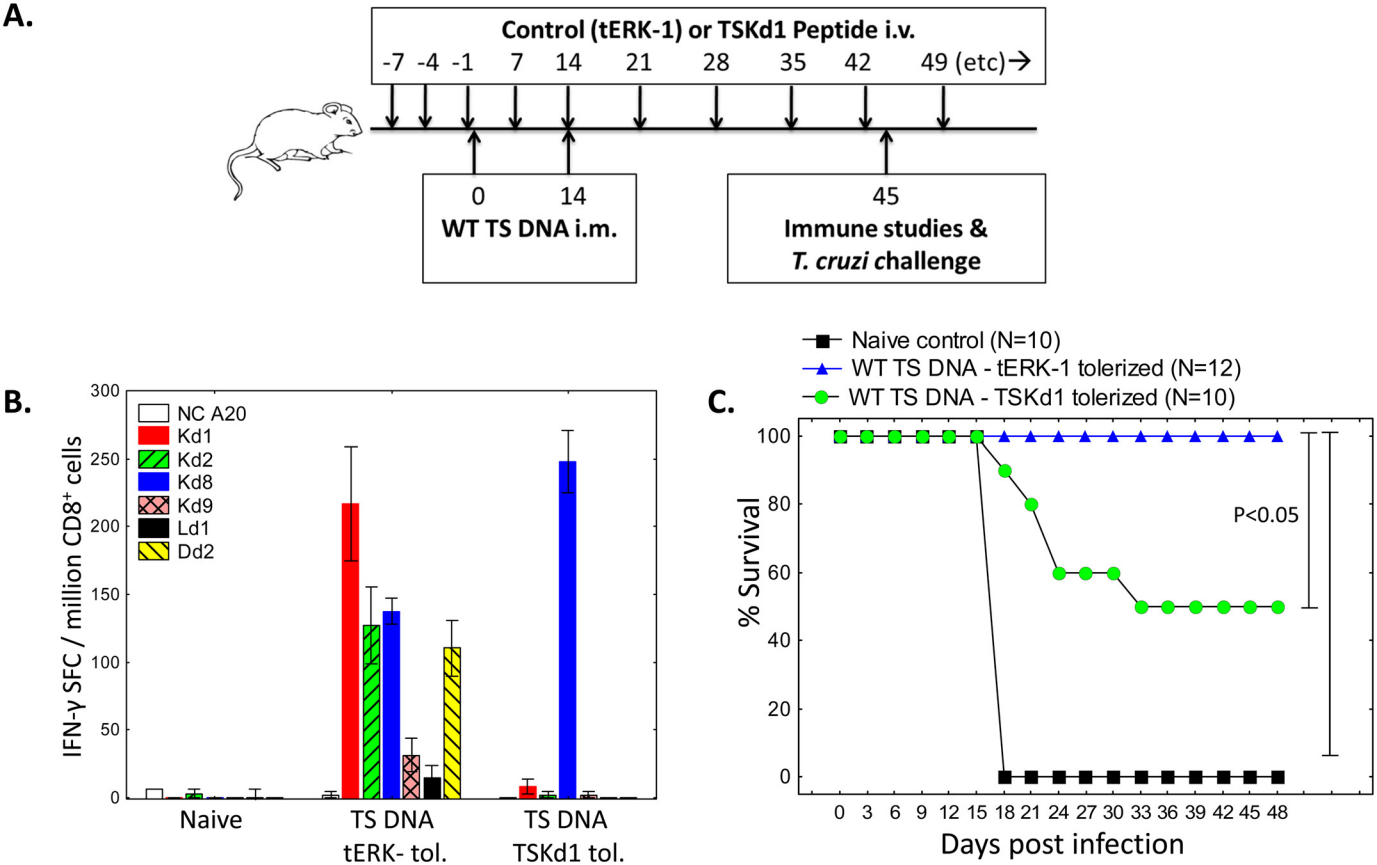
TSKd1 tolerization during WT TS DNA vaccination reduces CD8^+^ T cell responses directed against both immunodominant and subdominant TS epitopes. BALB/c mice were injected with tERK-1 control or TSKd1 peptide i.v. on indicated days before and after i.m. vaccination with WT TS DNA followed by *T*. *cruzi* challenge as shown in panel A. One month following the final immunization, CD8^+^ splenic T cells from representative mice were stimulated with APC (A20) pulsed with TS peptides in overnight IFN-γ ELISPOT assays (B). Results are representative of 2 independent experiments. Other groups of tolerized TS DNA vaccinated mice were challenged with 5,000 *T*. *cruzi* BFT and survival monitored (C; 10–12 mice/group; p<0.05 comparing TS DNA vaccinated tolerized with TSKd1 or the tERK-1 peptide by Fisher exact tests).

The in silico and immunoprecipitation experiments described above strongly indicate that similar TS antigen protein levels and structure are seen by the BALB/c murine immune system after immunization with the WT and TSKd1 null TS DNA vaccines. However, to further rule out major physical differences between the TS proteins as an explanation for the failure of the TSKd1 null vaccine to induce subdominant TS-specific CD8^+^ T cell epitopes, we studied the effects of TSKd1 tolerization during WT TS vaccination. Mice were vaccinated twice 2 weeks apart with the WT TS vaccine and injected intravenously pre- and post-vaccination with either the irrelevant control tERK-1 K^d^-restricted peptide [26], or TSKd1 (Fig 5A). CD8^+^ T cells from these mice were stimulated in vitro with a panel of immunodominant and subdominant TS peptides in IFN-γ ELISPOT assays. As expected, tolerization with the control tERK-1 peptide during WT TS vaccination did not alter induction of the complex immunodominant and subdominant epitope responses (Fig 5B). In contrast, tolerization with the TSKd1 peptide resulted in the same pattern of TS-specific epitope responses seen with the TSKd1 null vaccine (only TSKd8-specific responses were detectable). Immunization and challenge experiments confirmed that the more complex immunodominant/subdominant response pattern was significantly more protective (Fig 5C). This difference in protection again was detected despite a similar total number of T cells being induced by WT TS DNA vaccination in both TSKd1- and tERK-1-tolerized mice (S4 Fig). Thus, it is clear that the immunodominant TSKd1 epitope has immunodominant protective effects, associated with a paradoxical enhancement of TS-specific subdominant CD8^+^ T cell epitope responses.

### The subdominant epitope enhancing effects associated with WT TS DNA vaccination mechanistically involve CD45-mediated costimulatory activity

As shown in S3 Fig, the overall predicted tertiary structures of WT TS and TSKd1 null TS proteins are similar. In addition, the predicted structure and location of many of the amino acids shown to be important in enzymatic activity are unaffected by mutation of TSKd1 (i.e., the 3 arginine residues known as the arginine triad which bind carboxylate groups in sialic acid molecules; S5 Fig). However, the position and orientation of other amino acid side chains predicted within the enzymatic pocket (some of which have been previously implicated in binding of TS ligands) were altered (S5 Fig). It is possible that the minor positional changes in key amino acids within the catalytic pocket could have an effect on TS ligand binding and enzymatic activity.

**Fig 6:**
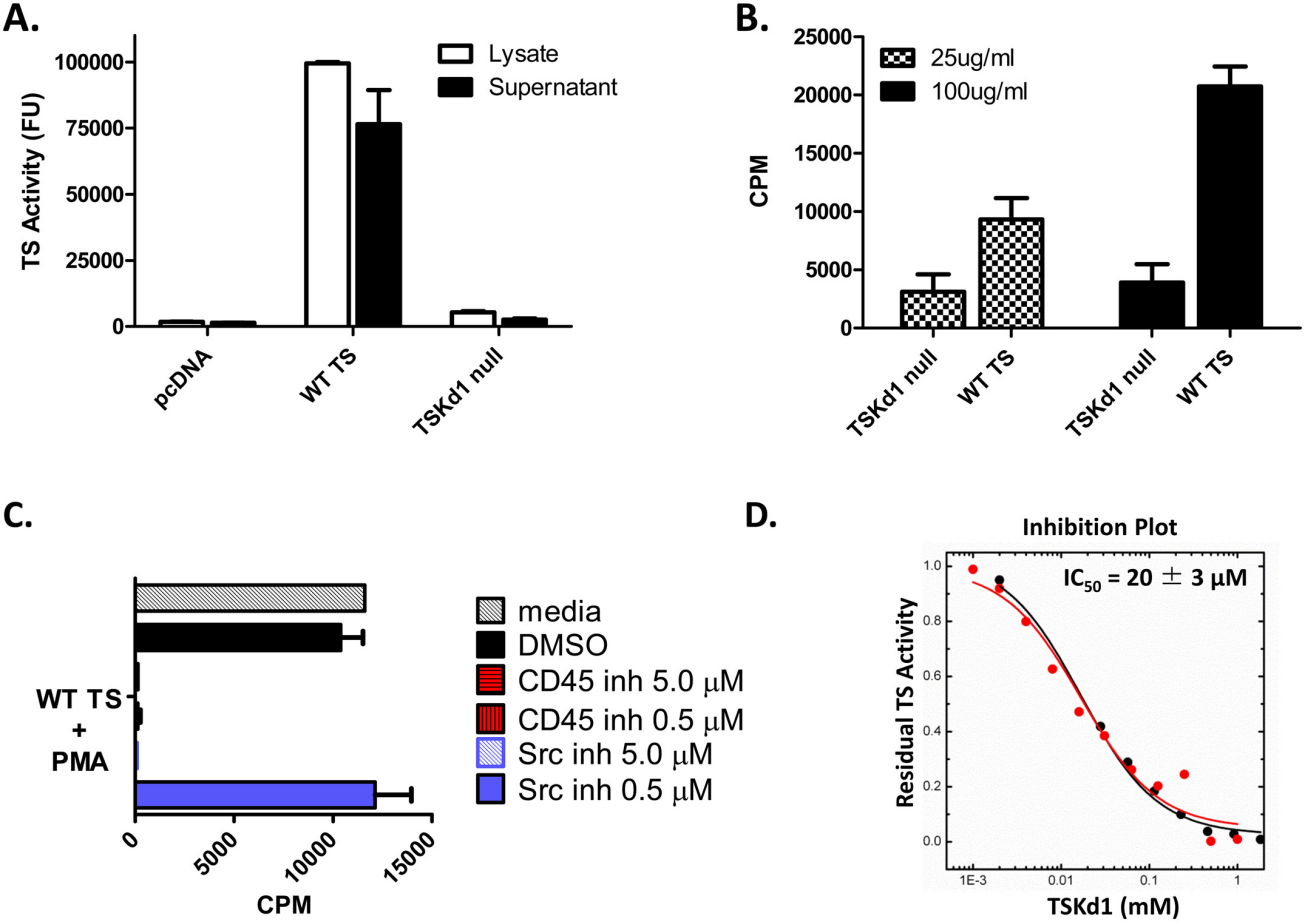
TS enzymatic activity is associated with costimulatory effects. In panel A, enzymatic activities of WT and TSKd1 mutant proteins were determined in lysates and supernatants of 293T cells transfected with WT TS and TSKd1 null DNA using fluorometric TS enzymatic activity assays. Panel B shows the co-stimulatory properties of WT rTS and TSKd1 null rTS on naïve CD8^+^ T cells. CD8^+^ T cells from naïve 4–5 week old BALB/c mice were purified by positive magnetic bead selection and incubated with suboptimal doses of PMA (12.5ng/ml) ± WT or TSKd1 null rTS. After 3 days, proliferation was measured by ^3^H-Thymidine incorporation. Shown are incorporated ^3^H-Thymidine counts per minute (CPM) above suboptimal PMA treatment alone. Similar TS costimulation assays using naïve CD8^+^ T cells were conducted with CD45 inhibitor PTP or Src-family kinase inhibitor PP2 added (C). In panel D, increasing concentrations of TSKd1 peptide were added to constant amounts of WT TS in TS enzyme assays. Results shown are representative of 2–4 independent experiments.

A previous report indicated that TS could costimulate CD4^+^ T cell responses due to the ability of the functional TS enzyme to bind sialylated substrates on mammalian T cells [e.g.-CD43, [27]]. Therefore, we next studied whether the proteins encoded by the WT TS and TSKd1 null TS DNA vaccines differed in functional enzymatic activity. Fig 6A demonstrates that only supernatants and lysates from 293T cells transfected with WT TS, and not supernatants and lysates of cells transfected with TSKd1 null TS, expressed trans-sialidase enzymatic activity.

To address whether or not WT TS but not TSKd1 null TS might induce CD8^+^ T cell activation, we stimulated naïve CD8^+^ T cells from BALB/c mice with suboptimal doses of PMA in the presence of purified WT or TSKd1 null TS and measured CD8^+^ T cell proliferation after 3 days. Indeed WT TS provided significantly higher costimulatory activity compared to TSKd1 null TS (Fig 6B). Because TS had been shown to costimulate CD4^+^ T cell responses by signaling through CD43 we compared the costimulatory effects of WT TS on WT and CD43 KO CD8^+^ T cells. WT TS resulted in similar co-stimulation of both WT and CD43 KO CD8^+^ T cells (S6 Fig). Also, TS has been shown to induce B cell activation mediated through the cell surface mucin CD45. [28] Therefore, we next investigated whether the costimulatory activity of TS on CD8^+^ T cells required CD45 signaling, adding CD45 or Src-family kinase inhibitors (PTP and PP2, respectively) to TS/PMA activated CD8^+^ T cell cultures. TS costimulatory activity was blocked by addition of both CD45 and Src-family kinase inhibitors (Fig 6C), indicating that TS costimulatory activity on CD8^+^ T cells is dependent on CD45-mediated signaling. This mechanism of CD45 mediated costimulation of CD8^+^ T cells can explain the more complex induction of subdominant TS-specific CD8^+^ T cell responses by the WT enzymatically active TS vaccine.

These overall results led us to hypothesize that in the tolerization experiments presented above, the high doses of the tolerizing TSKd1 peptide given intravenously could inhibit the costimulatory activity of WT TS. To test this hypothesis, we next investigated whether the immunodominant epitope TSKd1 could have an inhibitory effect on the enzymatic activity of TS in vitro. Increasing amounts of purified TSKd1 peptide were added to a constant dose of WT TS enzyme, and indeed, the enzymatic activity of WT TS was significantly inhibited by addition of the TSKd1 CD8^+^ T cell epitope (Fig 6D; IC_50_ = 20 ± 3 μM).

### The subdominant epitope enhancing effects associated with TSKd1 are counterproductive for host immunity

**Fig 7:**
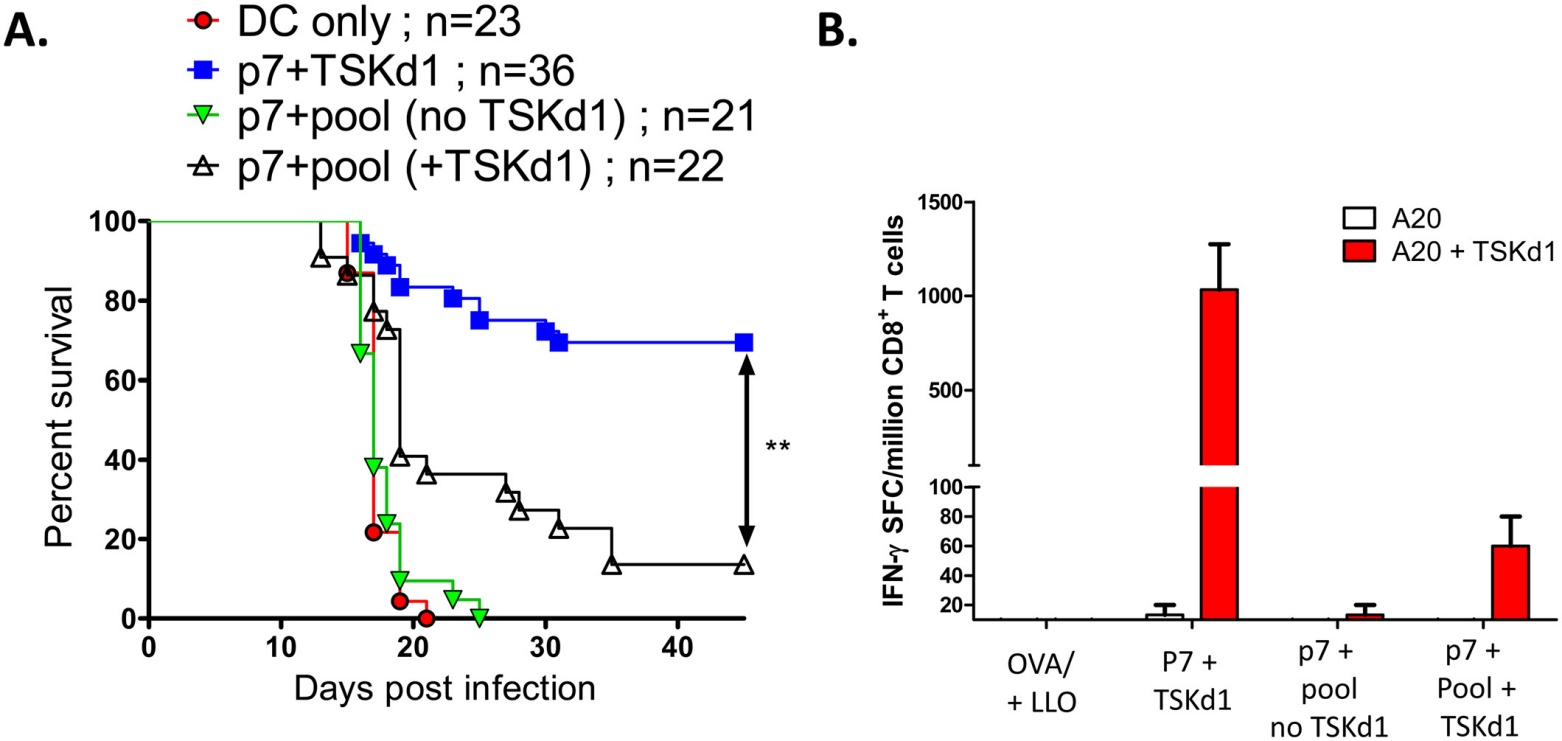
The WT TS vaccine-induced subdominant epitope responses are counterproductive for host immunity. B16-Flt3L-induced and in vivo LPS matured CD11c^+^ dendritic cells were pulsed with TS peptides and injected i.v. into groups of naïve BALB/c mice (1x10^6^ DC, twice, 2 weeks apart). The peptide pool was comprised of the panel of K^d^, D^d^, and L^d^-restricted epitopes differentially induced by WT TS and TSKd1 null TS vaccination (TS peptides Kd2, Kd5, Kd6, Kd7, Kd9, Ld1, and Dd2). Groups of control DC (no peptide) and TS peptide-pulsed DC vaccinated mice were challenged 4 weeks later with 5,000 *T*. *cruzi* BFT and survival monitored (A). Mice vaccinated with p7 + TSKd1 alone were significantly more protected than mice vaccinated with p7 + pool (+ TSKd1) by Log-Rank [Mantel-Cox] Test, p<0.0001. Shown are cumulative results from multiple independent experiments. In addition, CD8^+^ T cells from representative vaccinated mice harvested pre-challenge (one month following the final DC immunization) were stimulated in IFN-γ ELISPOT assays with APC (A20) pulsed with the immunodominant TSKd1 peptide (B).

The above results indicate that WT TS costimulatory activity could mechanistically explain why WT and not TSKd1 null TS vaccines have enhancing activity for TS-specific subdominant CD8^+^ T cell epitopes. However, none of our previous studies addressed whether the TS subdominant CD8^+^ T cell responses were beneficial or detrimental for host immunity. With the DC vaccination model used above, we were able to address this latter question. DC were pulsed with the CD4 TS epitope p7 and the TSKd1 epitope alone, or with these epitopes plus a pool of subdominant epitopes, and used to vaccinate BALB/c mice. These experiments clearly demonstrated that mice vaccinated with the p7 and TSKd1 epitopes alone were significantly better protected against systemic virulent *T*. *cruzi* challenges, than mice vaccinated with p7, TSKd1 and the pool of subdominant epitopes (Fig 7A; *p<0.0001 by Log-Rank [Mantel-Cox] Test and Fisher exact, 2-tailed test). Furthermore, mice vaccinated with p7 plus the pool of subdominant epitopes alone (without the TSKd1 epitope) were as susceptible to *T*. *cruzi* challenge as controls vaccinated with DC but no peptides (Fig 7A), and the addition of the subdominant epitopes was shown to inhibit the induction of TSKd1-specific CD8^+^ T cell responses (Fig 7B). These results suggest that this mechanism of paradoxical induction of both immunodominant protective and subdominant non-protective CD8^+^ T cell responses may have evolved as a mechanism advantageous for the parasite, counterproductive for the development of optimal host immunity.

## Discussion

The *T*. *cruzi* protozoan parasite causes Chagas Disease which affects 10–12 million people. Chronic intracellular infection is controlled but not eliminated by immune responses, and both Chagasic cardiac and gastrointestinal disease occur after decades of parasite-directed immune inflammation. How the parasite eludes immune clearance and persists for the life of the infected individual in the absence of chemotherapeutic cure is unknown.

The large trans-sialidase (TS) superfamily of homologous *T*. *cruzi* proteins includes immunodominant antigens capable of inducing protective memory immune responses. [8, 9, 17] Furthermore, in both humans and mice infected with *T*. *cruzi*, CD8^+^ T cells specific for individual TS-encoded epitopes can achieve very high circulating frequencies essentially representing the majority of the immune response mounted against this complex parasitic pathogen. [17, 24] However, the accumulation of hundreds of related TS genes within the *T*. *cruzi* parasite genome suggests that this superfamily has evolved as an immunoevasive strategy critical for parasite persistence. Similar, but nonidentical TS-encoded T cell epitopes could function as altered peptide ligands, dampening the protective effector functions of individual TS-specific T cells. [29, 30] This speculated immunoevasion mechanism might suggest that TS antigens should not be included in *T*. *cruzi* vaccine candidates, although no direct evidence for TS-related immunoevasion has been demonstrated, and multiple investigators have shown that TS vaccines can induce potent immune responses protective against acute *T*. *cruzi* mucosal and systemic challenges at least as efficacious as any other parasite antigen investigated so far. [9, 18, 19]

TS enzymatic activity is essential for parasite virulence [12–14], TS genes encoding TS enzymatic activity are >95% identical and highly conserved among all *T*. *cruzi* isolates [16, 31], and both mice and humans develop potent TS-specific immune responses during natural infections. [12, 24, 25, 32] Therefore, we have been investigating the consensus TS enzymatic domain as a potential vaccine candidate. However, in view of the potential for TS-specific immune responses to be targeted by an evolutionarily derived immunoevasion strategy, we have now sought to fully characterize the molecular detail of immune responses induced by our TS candidate vaccines in a murine model to further direct the development of optimal prophylactic and immunotherapeutic vaccines.

We have previously identified an I-A^d^-restricted CD4 epitope encoded within the consensus TS enzymatic domain that can provide the T cell helper functions required to induce optimal protective immunity. We also identified a single immunodominant K^d^-restricted CD8 peptide encoded within the consensus TS enzymatic domain that is required for the induction of optimal protective immunity. We further demonstrated that this single pair of TS-specific CD4 and CD8 epitopes is sufficient for induction of protective immunity in BALB/c mice (Figs 1B, 1C and 7A). The identification of these important immunodominant and protective T cell epitopes has allowed us to characterize the molecular detail of the immune networks induced by our TS vaccines. We found that CD8^+^ T cell responses induced by the intact, full-length consensus and enzymatically active TS domain are complex, involving numerous subdominant epitope responses restricted by H-2K^d^, H-2D^d^ and H-2L^d^.

It was surprising that the responses directed against both the immunodominant and subdominant TS-specific CD8 epitopes were induced in parallel, because immunodominance has commonly been associated with the suppression of subdominant T cell responses. Also unexpected was the finding that the overall immune responses associated with this broadening of TS epitope specificity were less protective than responses directed against only the immunodominant epitopes. DC vaccines pulsed with the immunodominant TSKd1 CD8 T cell epitope alone were significantly more protective than DC vaccines pulsed with a combination of TS-specific immunodominant and subdominant CD8 T cell epitopes (Fig 7A).

Both dominant and subdominant CD8^+^ T cell responses were induced by the WT enzymatically active TS antigen, and induction of subdominant responses required T cell costimulatory function mediated through CD45 (Fig 6A–6C). Costimulatory effects have been attributed to TS previously, and were thought to be mediated by TS signaling through CD43, a heavily sialylated known surface costimulatory molecule. [27] We obtained CD43 knockout mice but were unable to demonstrate a failure of costimulatory activity in CD8^+^ T cells obtained from these CD43 KO mice with our enzymatically active TS antigen (S6 Fig). Differences between our costimulatory studies and those reported previously included the use of different mouse strains (BALB/c in our work compared with C57BL/6 in the previous work) and studies of different T cell subsets (CD8^+^ T cells in our work compared with CD4^+^ T cells in the previous work). It is possible that TS can trigger costimulatory signals in CD8^+^ BALB/c T cells and CD4^+^ C57BL/6 T cells via different sialylated surface molecules (or acceptor molecules) competent to be sialylated by TS activity. The observed enhancing effects of WT TS on TS-specific antibody responses (Fig 4D) are likely due to the previously described costimulatory activity of TS for CD4^+^ T cell activation, leading to increased helper T cell effects on B cell responses.

Surprisingly, we found that if we removed the MHC anchoring positions in the TSKd1 immunodominant CD8^+^ T cell epitope, we not only prevented immunodominant T cell responses from being induced, but in addition found that subdominant CD8^+^ T cell responses directed against multiple other TS-encoded subdominant T cell epitopes were abolished. Similar amounts of TS protein were expressed by both WT and mutant TS vaccines, and the overall structural features of both expressed antigens were similar indicating that the 2 vaccine antigens were unlikely to be processed differently by APCs. Furthermore, similar overall and TSKd8-specific CD8^+^ T cell responses were induced by both vaccines confirming that differences in protein expression or antigen processing were not responsible for the differences in subdominant epitope immunogenicity displayed by the 2 vaccines. We believe the loss of the subdominant enhancing effects in this site-directed mutagenesis model can be explained by the fact that the mutations introduced led to enzymatically inactive TS protein which could not provide the required costimulatory effects. The structural modeling studies presented in S3 and S5 Figs do show a minor structural change in the TSKd1 null protein that likely explains the phenomenon.

In our model of TSKd1 tolerization during WT TS DNA vaccination we observed a similar alteration in the pattern of subdominant TS-specific CD8^+^ T cell responses induced. In this case, the vaccine antigen was identical, and therefore differences in protein expression or antigen processing were not possible. To explain these results, we initially hypothesized that TS could indirectly enhance subdominant epitope responses by either increasing APC function, or by TSKd1-specific T cell production of T cell activating cytokines, and that TSKd1 tolerization could prevent these effects. However, the results presented in Fig 6B demonstrate costimulatory activity of WT TS in an APC-free in vitro system without the addition of TSKd1-specific T cells. An alternative explanation for the TSKd1 tolerization protocol interfering with the induction of subdominant responses is that intravenous injection of high doses of the TSKd1 peptide during TS vaccination can interfere with the costimulatory activity mediated by substrate binding enzymatically active TS antigen (e.g.-by blocking TS sialylation of key costimulatory molecules such as CD45 expressed on the surface of CD8^+^ T cells). We have made exploratory attempts to block rTS-induced CD45-mediated costimulation of naïve CD8^+^ T cells with soluble TSKd1 peptide without effect. However, we have shown that soluble TSKd1 peptide interacts with recombinant TS in controlled enzyme assays, preventing enzymatic activity of wild type TS (Fig 6D). Future studies directed to more fully elucidate the biochemical interactions and biologic effects of TSKd1 peptide interference with TS enzymatic activity and TS costimulatory effects on CD8^+^ T cells are thus needed.

TS has been shown to sialylate host cell glycoproteins including CD45 present on both T and B cells. [28, 33] CD45 is an important signaling molecule which acts as a rheostat controlling B and T cell receptor sensitivity. TS induces IL-17 production in activated B cells independent of the IL-17 transcription factors RORγt and Ahr. [28] Instead, these investigators showed that IL-17 production was driven through CD45- and Btk-dependent signaling. It is likely that TS sialylation of CD45 signals through a similar fashion in T cells. We have demonstrated that WT enzymatically active TS, but not the TSKd1 null mutated inactive TS, acts as a co-stimulant for naïve CD8^+^ T cells (Fig 6B). Furthermore, we have shown that PTP (the CD45 selective CD45 inhibitor) or PP2 (the Src-family inhibitor preventing phosphorylation of Lck and Fyn) prevent co-stimulation of naïve CD8^+^ T cells (Fig 6C). Thus it is likely that TS enzymatic modulation of CD45 and downstream activation of Src family kinases Lck and Fyn reduce the threshold for TCR activation of naïve T cells, leading to the increased responses to subdominant TS epitopes.

The costimulatory effects of enzymatically active TS could enhance the responses to a multitude of TS and non-TS *T*. *cruzi*-specific T cells counterproductive for optimal immunity during natural *T*. *cruzi* infection. These effects could have detrimental effects for host immunity and even be crucial for the establishment of chronic parasite infection. An important future direction will be to determine if selective epitope vaccines containing TS and/or other epitopes without enzymatically active TS antigen, or full-length TS antigens engineered to express key immunodominant epitopes but lacking the costimulatory helper effects for nonprotective subdominant epitope-specific responses, could induce more effective protective *T*. *cruzi* immunity.

We have shown that enzymatically active WT TS, but not inactive TSKd1 null TS, provides CD8^+^ T cell costimulatory function (Fig 6B). We observed robust proliferation of naïve CD8^+^ T cells with the lowest WT TS dose tested (25μg/ml). It is noted that this same concentration of TS may not be produced in vivo after TS DNA vaccination. It is likely that the transduced APC:T cell microenvironment plays an important role in costimulation of CD8^+^ T cells in vivo. It would be interesting to quantify the amounts of protein production within specific microenvironments after DNA vaccination for further mechanistic elucidation of the biologic effects reported here.

In summary, we have identified an absolutely unique subdominant CD8^+^ T cell enhancing phenomenon expressed by an important human pathogen that appears to increase the breadth of epitope-specific immune responses, but paradoxically prevents the development of optimal host immunity. Our data indicate that these unusual effects depend on an overly indiscriminant costimulatory activity associated with enzymatic trans-sialidase activity, and may represent an elegant evolutionary strategy developed for the purposes of immune escape/pathogen persistence. Further work is needed to study these effects in the context of the myriad of potential altered peptide ligands expressed by the complete TS gene superfamily. These results have important implications for the development of vaccines for Chagas disease, but also more broadly suggest that methods to enhance the breadth of epitope specificity induced by vaccines against other key pathogens need to be carefully studied before being applied for public health strategies in human populations.

## Materials and Methods

### Ethics statement

All animal studies were approved by the Institutional Animal Care and Use Committee (IACUC)/Animal Care Committee (ACC) at Saint Louis University (SLU IACUC Protocol #1106). The Saint Louis University IACUC provides oversight for compliance with all relevant laws and regulations so as to assist researchers, faculty, and students in the conduct of high quality research and teaching, thereby assuring the public of the humane care and use of vertebrate animals used for these endeavors. The Saint Louis University IACUC is mandated by the Animal Welfare Act (Public Law #89–544) and the Public Health Service (PHS) policy on Humane Care and Use of Laboratory Animals to evaluate the care, treatment, housing, and use of animals, and for certifying compliance with the Act by the research facility. Saint Louis University is a USDA registered research facility (Registration 43-R-011), is regularly inspected and files all required documentation, including an annual report. In addition, under the provisions of the Public Health Service Policy on the Humane Care and Use of Laboratory Animals, the University files required assurance documents to the Office of Laboratory Animal Welfare (OLAW; NIH Assurance A3225-01). The Animal Care and Use Program at Saint Louis University is fully accredited by the Association for Assessment and Accreditation of Laboratory Animal Care, International (AAALACi #000656, renewed July 2, 2015). CO_2_ from a compressed gas source was used to euthanize mice.

### Animals, parasites, and challenges

Six- to 8-wk-old female BALB/c mice were purchased from Charles River/NCI (Frederick, Maryland), and adult female New Zealand White rabbits were obtained from Myrtle’s Rabbitry (Thompson Station, TN). The Tulahuén strain of *T*. *cruzi* is a well characterized parasite strain with high virulence, and was passaged through BALB/c mice and *Dipetalogaster maximus* insects. Blood form trypomastigotes (BFT) were collected from infected mice, and challenges of 5000 BFT were given subcutaneously. All animal studies were conducted with the approval of the Saint Louis University Animal Care Committee in AALAC-accredited facilities.

### DNA vaccines

We used a DNA vaccine encoding the consensus TS enzymatic domain as described previously [9], originally provided by Dr. Mauricio Rodrigues. We generated a new TS DNA vaccine lacking a functional CD8 epitope (IYNVGQVSI, amino acids 359–367) [34] by site-directed mutagenesis (QuickChange II, Agilent Technologies, Inc.), altering the 2^nd^ position tyrosine to glycine and the 9^th^ position isoleucine to phenylalanine creating the “TSKd1 null” vaccine. These changes were predicted to completely abolish H-2K^d^ binding. We changed TAT to GGT (amino acid Y→G) and changed ATT to TTT (amino acid I → F). Plasmid DNA for vaccination was prepared using Qiagen EndoFree Giga-prep kits.

### Dendritic cell generation and vaccines

Splenic dendritic cells (DC) were isolated after intraperitoneal injection of BALB/c mice with 5x10^6^ B16 cells expressing Flt-3 ligand (provided by Dr. John Harty, University of Iowa) as previously described [35]. DC were induced to mature in vivo with LPS (2 μg i.v.) and harvested from spleens 16 hours later using anti-CD11c microbeads (Miltenyi Biotec, Inc.). The purity and activation status of DC were determined by staining for CD11c, CD86 and MHC class II. Routinely, >90% pure CD11c^+^ DC were obtained with yields of approximately 15–20x10^6^ DC per mouse. DC were pulsed with peptides [50–100μg/ml of p7 ± 50–100μg/ml TSKd1 ± 100μg/ml of an equally distributed pool of 7 subdominant TS peptides (Kd2, Kd5, Kd6, Kd7, Kd9, Ld1, and Dd2)] for 90 min, washed and then i.v. injected into BALB/c mice (1x10^6^/mouse).

### Immunizations

BALB/c mice were immunized either intramuscularly with 100 μg plasmid DNA or intravenously with peptide pulsed DC 2 or 3 times, 1–2 weeks apart. One month after the final immunization, mice were challenged subcutaneously with 5000 BFT. Pre- and post-challenge mice were studied for TS-specific immune responses and followed for survival. Rabbits were vaccinated subcutaneously with purified TS [9] in Freund’s adjuvant to generate polyclonal TS-antisera.

### IFN-γ enzyme-linked immunospot (ELISPOT) assays

Millititer HA 96-well microtiter plates (Millipore) were coated with murine anti-IFN-γ mAb (clone R46A2; BD) and blocked with 10% FBS. Total spleen cells or purified CD8^+^ T cells were stimulated with control A20J cells, stably TS-transfected A20J cells (both provided by Dr. Maurício M. Rodrigues, Sao Paolo, Brazil) or A20J cells pulsed with peptides (2μM) in T cell media [10% FBS, 50% EHAA (Invitrogen), 37% RPMI, supplemented with penicillin, streptomycin, gentamycin L-Glutamine, and 2-mercaptoethanol] [10]. Numbers of IFN-γ-producing cells were detected with biotinylated anti-IFN-γ (BD-Pharmingen), streptavidin conjugated to horseradish peroxidase (HRP) (Jackson Immunoresearch Laboratories) and 3-amino-9-ethylcarbazole substrate precipitation. The numbers of spot-forming cells (SFC) per million cells are reported.

### Transfections, immunoprecipitations, western blots, and ELISA

293T cells (ATCC, Manassas, VA) were cultured in 6-well plates (0.5x10^6^/ well) in 10% FBS-DMEM, and after 24 hours, cells were transfected with plasmid DNA mixed with FuGENE 6 (Roche) and cultured for 48 hours. Cell monolayers were solubilized with lysis buffer (20 mM Tris-HCl pH 7.5, 150 mM NaCl, 1 mM EDTA, 1 mM EGTA, 1% Triton X-100, 2.5 mM sodium pyrophosphate, 1 mM beta-glycerophosphate, 1 mM Na_3_VO_4_, 1 μg/ml leupeptin, and 1 mM phenylmethylsulfonyl fluoride) on ice for 10 min, cleared by centrifugation (12,000*g* for 10min at 4°C), and pre-absorbed with protein G plus/protein A agarose suspension (CALBIOCHEM). Pre-absorbed lysate was mixed with serum from a TS-immunized rabbit or with serum samples from TS DNA-vaccinated BALB/c mice at 4°C overnight. Immune complexes were precipitated with Protein G plus Protein A Agarose for 2 hours at 4°C, washed with lysis buffer, and resuspended in SDS–sample buffer for SDS-PAGE and Western blotting analysis. Total cell lysates (30–50 μg of protein) were run as positive controls. Western blots were incubated with the relevant primary antibodies, followed by incubation with HRP-conjugated secondary antibody, and bands visualized by ECL (Thermo scientific). TS-specific antibody responses in TS vaccinated mice were evaluated by ELISA as previously described. [11]

### Peptide-specific tolerance induction

Peptides were synthesized by Sigma Genosys or JPT Peptide Technologies GmbH. Lyophilized peptides were suspended in dimethyl sulfoxide (DMSO) at 100 mg/ml and stored at -20°C. 300 μg of each tolerizing peptide diluted in PBS was i.v. administered 7 days before vaccination, and 100 μg administered at 4 days and 1 day pre-vaccination and weekly until the end of the experiments. Representative mice were sacrificed 7 days after the final peptide treatment for immune studies. The remaining mice were challenged with 5000 BFT 1 month after the final vaccination for survival studies.

### Trans-sialidase enzymatic activity measurements

Trans-sialidase enzymatic activity was assayed as described previously [36]. Briefly, trans-sialidase purified proteins or transfected cell lysates were mixed with 1 mM 3’sialyllactose (SL), 0.5 mM 4-methylumbelliferyl-β-D-galactoside (MUGal) in 50 μl of 100 PIPES (pH 7), and incubated at 20°C for 45 minutes. After stopping the trans-sialidase reactions with 1 ml of ice cold water, samples were added to acetate-treated Q-Sepharose and sialylated 4-methylumbelliferyl-β-D-sialylgalactoside product (MUGalNeuAc) eluted with 1 M HCl. The acid elutions were incubated at 95°C for 45 minutes, the reactions neutralized with glycine/NaOH (pH 10) and released 4-MU measured by fluorescence emission at 450nm and compared to a standard curve. Inhibition of the enzymatic activity of TS in the presence TSKd1 was assayed by following the release of the fluorescent product 4-methylumbrelliferone (Mu) from the substrate MuNANA. Excitation and emission wavelengths were 322 and 448 nm, respectively. The reaction was continuously monitored in a SpectraMax i3 Multi-Mode Detection Platform (Molecular Devices) after pre-incubation of the enzyme with the peptide for 10 min at room temperature. The residual activity at each concentration of TSKd1 was obtained by dividing the slope calculated at each concentration of TSKd1 (V_i_) by the initial velocity determined in the absence of peptide (V_0_). The concentration-response data fit to a simple binding isotherm equation with best-fit parameter for the IC_50_ value.

### CD8^+^ T cell costimulation assay

CD8^+^ T cells purified by positive Miltenyi magnetic bead selection from naïve 4–5 week old BALB/c mice were added to 96-well flat bottom plates (2x10^5^ cells/well) and stimulated with suboptimal doses of PMA (12.5 ng/ml) with or without recombinant WT vs TSKd1 null TS protein (25–100 μg/ml) similar to TS costimulatory assays conducted previously [27]. Three days later, 0.5 μCi/well of ^3^H-thymidine was added, and after 6 more hours, cells were harvested onto glass fiber filtermats. Ultima Gold F scintillation fluid was added and filtermats counted using a Perkin Elmer TriLux MicroBeta 1450 instrument. The CD45 PTP inhibitor N-[(9-10-dioxo-9,10-dihydro-phenanthren-2-yl)-2,2-dimethyl-propionamide] and Src-family inhibitor PP2 [4-amino-5-(4-chlorophenyl)-7-(t-butyl)pyrazolo[3,4-d]pyrimidine] (Millipore EMD) were found to be non-cytotoxic to PMA-activated CD8^+^ T cells at inhibitory concentrations (S7 Fig).

## Supporting Information

S1 Table MHC (H2-K^d^, D^d^, and L^d^) binding predictions of the TS protein sequence. For each of the 5 prediction tools shown below, potential binders were ranked and assigned scores from 1–11 (top 10 predicted binders scored 1–10, and all others assigned value of 11; data acquired March, 2008). These scores were then averaged, and the top 9 H2-K^d^, 7 H2-D^d^, and 5 H2-L^d^ predicted binders are shown. Bimas: accessed at http://www-bimas.cit.nih.gov/molbio/hla_bind/. Syfpeithi: accessed at http://www.syfpeithi.de/Scripts/MHCServer.dll/EpitopePrediction.htm; currently available at http://www.syfpeithi.de/bin/MHCServer.dll/EpitopePrediction.htm. Rankpep: accessed at http://bio.dfci.harvard.edu/RANKPEP (No longer available;PMID12175724). IEDB: accessed at http://tools.immuneepitope.org/. Pred BALBc: accessed at http://antigen.i2r.a-star.edu.sg/predBalbc/; currently available at http://cvc.dfci.harvard.edu/balbc/
(TIF)

S1 Fig Vaccination of mice with WT TS and TSKd1 null DNA induces similar overall TS-specific IFN-γ producing T cell responses. BALB/c mice were immunized twice 2 weeks apart with WT TS or TSKd1 null DNA vaccines. One month later, total spleen cells were obtained and studied in IFN-γ ELISPOT assays with APC (A20) transfected with the full-length catalytic domain of TS (TS A20) or pulsed with the immunodominant TSKd1 peptide.
(TIF)

S2 Fig Vaccination with WT TS and TSKd1 null DNA induces similar TS-specific T cell lymphoproliferative responses. BALB/c mice were immunized i.m. with 100μg WT TS or TSKd1 null DNA twice, 2 weeks apart. Spleen cells obtained one month after the final vaccination were stimulated with wild type recombinant TS (rTS) and proliferation was assessed after 3 days of culture using ^3^H-thymidine incorporation assays.
(TIF)

S3 Fig Structural similarity between wild type TS and TSKd1 null TS proteins. In panel A, lysates were prepared from 293T cells transfected with negative control pcDNA (NC), wild type TS DNA (WT TS), or TSKd1 null DNA, and immunoprecipitated with pooled serum samples obtained from wild type TS DNA or TSKd1 null vaccinated mice. TS-specific western blots were then performed with the immunoprecipitates. Purified rTS immunoprecipitated using rabbit α-TS served as the positive control. Serum obtained from both WT TS and TSKd1 null vaccinated mice pulled down both homologous and heterologous TS proteins, further supporting similar tertiary structures of wild type and TSKd1 null TS proteins. Deduced amino acid sequences of wild type TS and the TSKd1 null constructs were utilized to create structural 3D models using BioLuminate (Version 1.7, Schrödinger, LLC, New York, NY) and PyMOL (PyMOL Molecular Graphics System, Version 1.7.4 Schrödinger, LLC) as shown in (B).
(TIF)

S4 Fig WT TS vaccinated mice tolerized against TSKd1 or tERK-1 developed similar overall TS-specific IFN-γ responses. Large quantities of peptide (tERK-1 control and TSKd1, 100–300μg/dose) were injected i.v into BALB/c mice starting one week prior to wild type TS DNA vaccination (peptide i.v. on days -7, -3, -1, 7, 14, 21, 28, and 35 in relation to first TS DNA vaccination). Four weeks after the second and final immunization, spleen cells were removed and stimulated with APC (A20) transfected with the full length TS catalytic domain in overnight IFN-γ ELISPOT assays.
(TIF)

S5 Fig Comparison of WT TS and TSKd1 null TS catalytic domain predicted structures. Structural models of wild type TS (green) and the TSKd1 null (magenta) constructs were created using BioLuminate and PyMOL. Highlighted in panel A are the 2 amino acids mutated to create the TSKd1 null vaccine (red and orange spheres represent the Y360G and I367F mutations, respectively). The black spheres depict the 3 arginine residues (R67, R277 and R346) which compromise the arginine triad important in binding TS substrates. In panel B, a merged enlarged view of the catalytic pocket predicted within WT TS and TSKd1 null TS is shown. Amino acid side chains of the 3 arginine residues (R67, R277, and R346) as well as 2 amino acids seemingly altered by mutation of TSKd1 (Y374 and D91) are noted.
(TIF)

S6 Fig Costimulatory effects of enzymatically active WT TS are not mediated through CD43. Naïve wild type (WT) B6 and naïve CD43^-/-^ CD8+ T cells were purified by positive magnetic bead selection and incubated with suboptimal doses of PMA (12.5ng/ml) ± WT rTS (25–100μg/ml). After 3 days, proliferation was measured by 3H-Thymidine incorporation.
(TIF)

S7 Fig CD45 and Src-family kinase inhibitors are not cytotoxic to PMA-activated CD8^+^ T cells. Naïve wild type BALB/c CD8^+^ T cells were purified by positive magnetic bead selection and incubated with suboptimal doses of PMA (12.5ng/ml) in the presence or absence of the indicated concentrations of CD45 inhibitor PTP or Src-family kinase inhibitor PP2. After 2 days of culture, cell viability was assessed by trypan blue exclusion microscopy.
(TIF)
