# Supplementary figures and images for: Costimulatory Effects of an Immunodominant Parasite Antigen Paradoxically Prevent Induction of Optimal CD8 T Cell Protective Immunity
Source: PLoS Pathog. 2016 Sep 19;12(9):e1005896. doi: 10.1371/journal.ppat.1005896 (PMC5028030; doi:10.1371/journal.ppat.1005896)

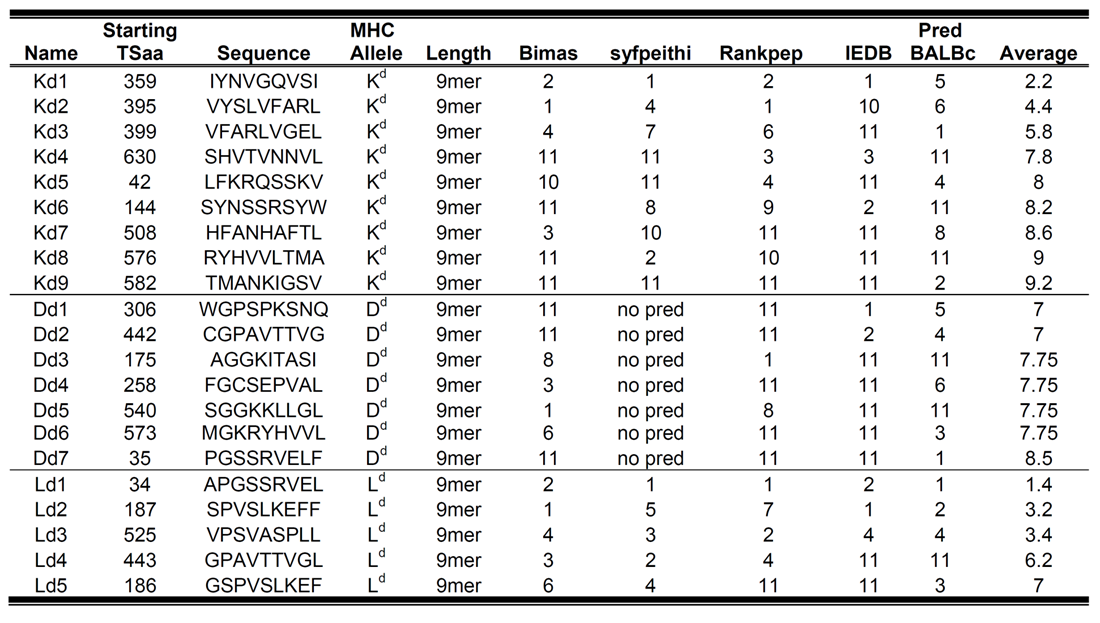

Supplement: S1 Table — For each of the 5 prediction tools shown below, potential binders were ranked and assigned scores from 1–11 (top 10 predicted binders scored 1–10, and all others assigned value of 11; data acquired March, 2008). These scores were then averaged, and the top 9 H2-Kd, 7 H2-Dd, and 5 H2-Ld predicted binders are shown. Bimas: accessed at http://www-bimas.cit.nih.gov/molbio/hla_bind/. Syfpeithi: accessed at http://www.syfpeithi.de/Scripts/MHCServer.dll/EpitopePrediction.htm; currently available at http://www.syfpeithi.de/bin/MHCServer.dll/EpitopePrediction.htm. Rankpep: accessed at http://bio.dfci.harvard.edu/RANKPEP (No longer available;PMID12175724). IEDB: accessed at http://tools.immuneepitope.org/. Pred BALBc: accessed at http://antigen.i2r.a-star.edu.sg/predBalbc/; currently available at http://cvc.dfci.harvard.edu/balbc/ (TIF) [file ppat.1005896.s001.tif]

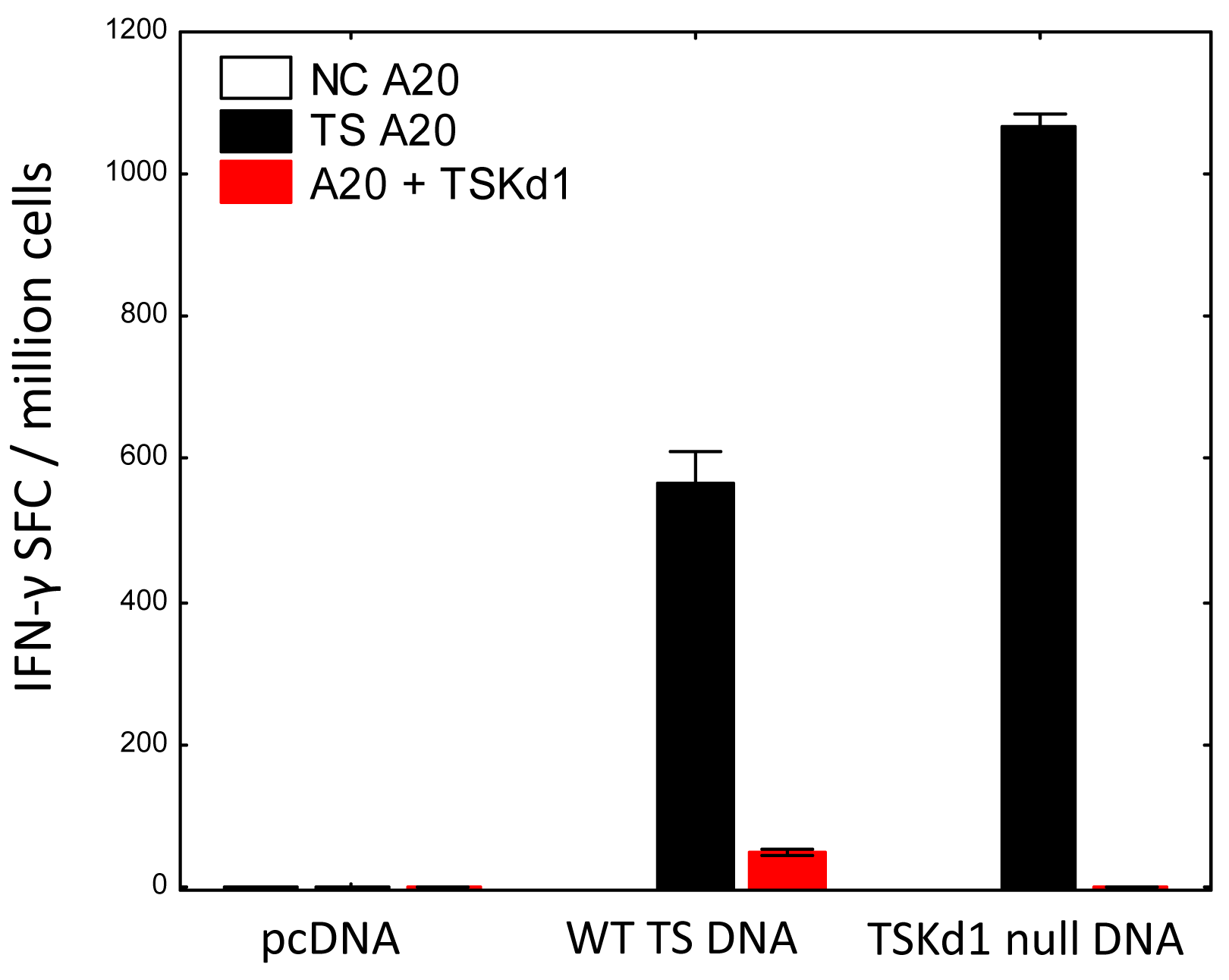

Supplement: S1 Fig — BALB/c mice were immunized twice 2 weeks apart with WT TS or TSKd1 null DNA vaccines. One month later, total spleen cells were obtained and studied in IFN-γ ELISPOT assays with APC (A20) transfected with the full-length catalytic domain of TS (TS A20) or pulsed with the immunodominant TSKd1 peptide. (TIF) [file ppat.1005896.s002.tif]

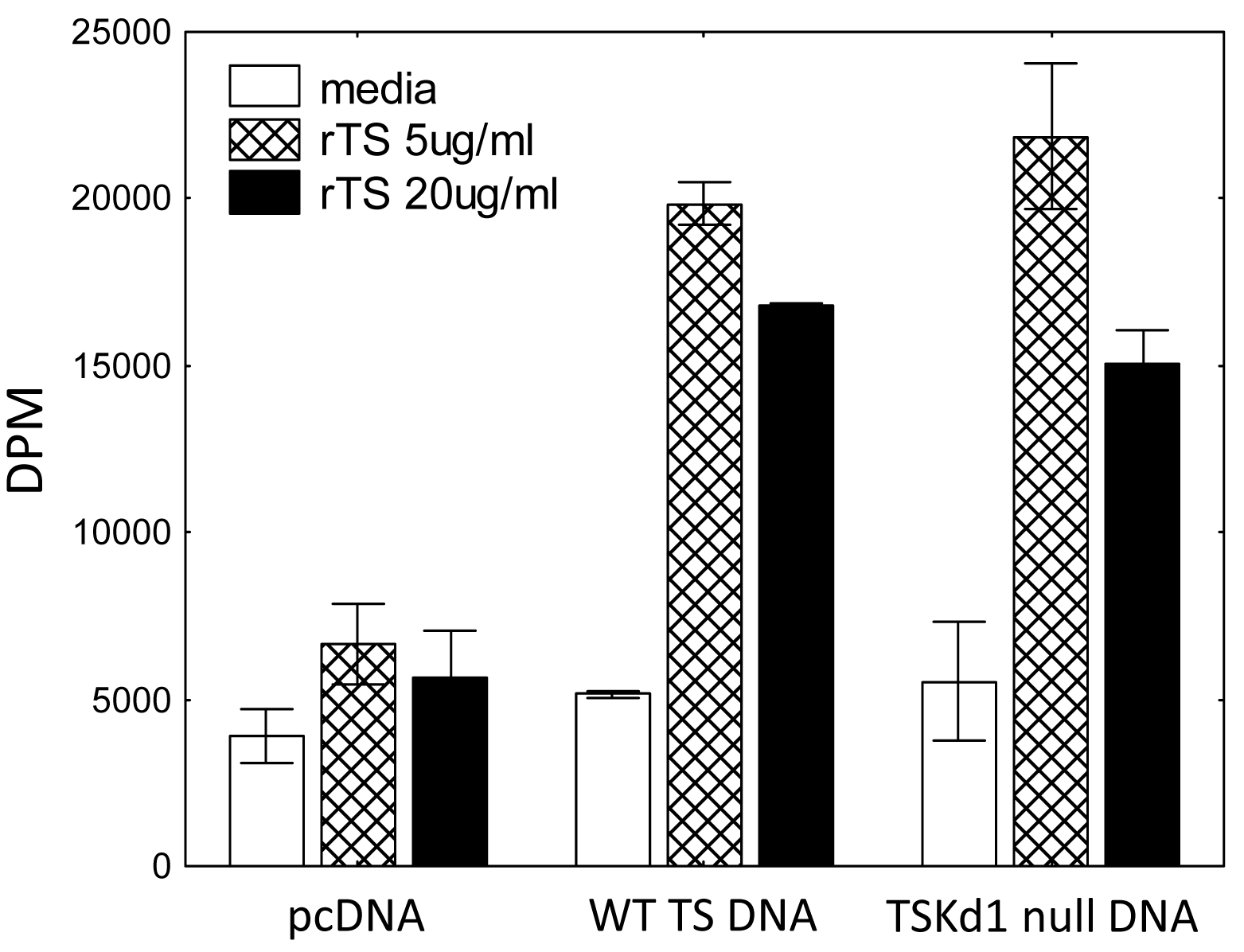

Supplement: S2 Fig — BALB/c mice were immunized i.m. with 100μg WT TS or TSKd1 null DNA twice, 2 weeks apart. Spleen cells obtained one month after the final vaccination were stimulated with wild type recombinant TS (rTS) and proliferation was assessed after 3 days of culture using 3H-thymidine incorporation assays. (TIF) [file ppat.1005896.s003.tif]

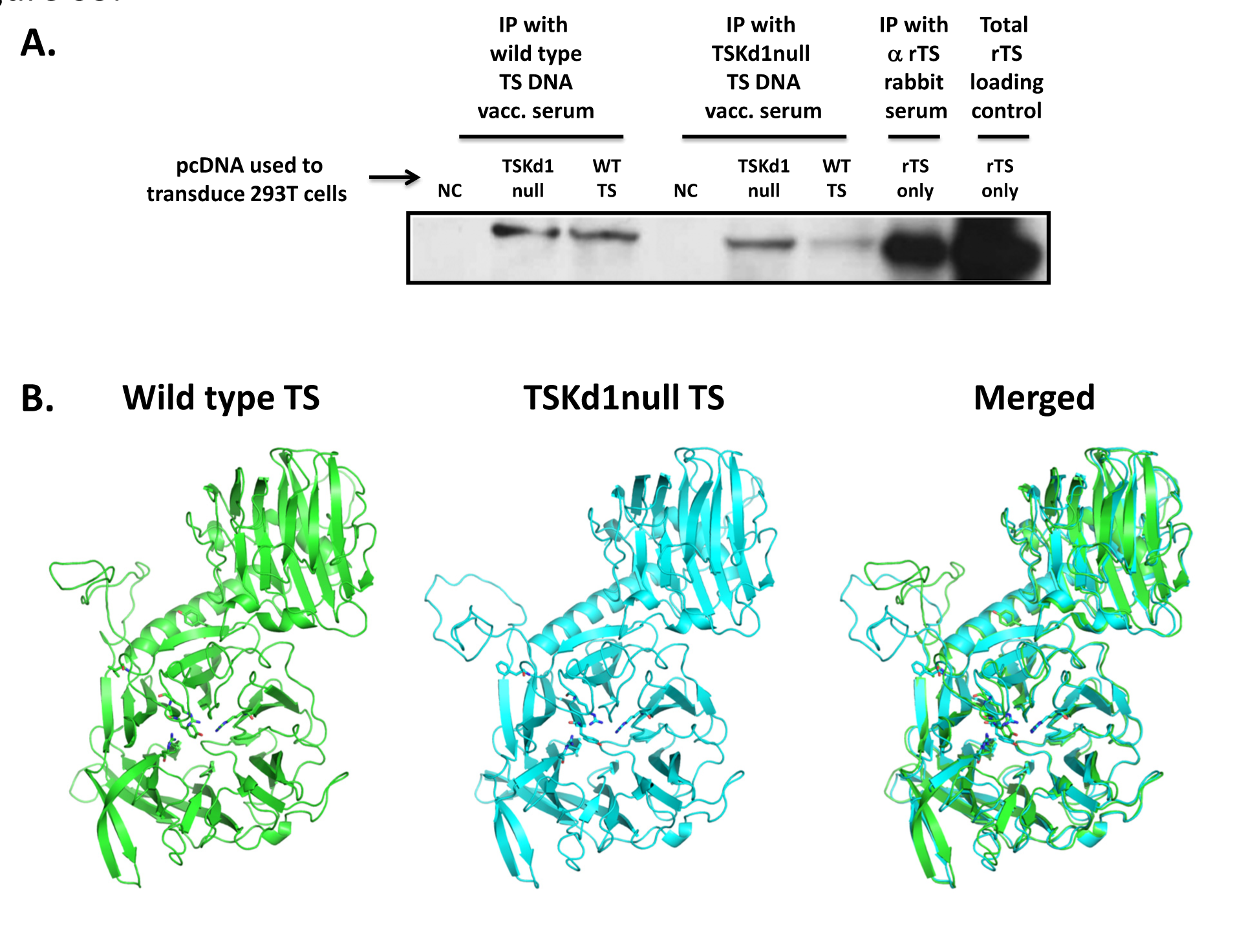

Supplement: S3 Fig — In panel A, lysates were prepared from 293T cells transfected with negative control pcDNA (NC), wild type TS DNA (WT TS), or TSKd1 null DNA, and immunoprecipitated with pooled serum samples obtained from wild type TS DNA or TSKd1 null vaccinated mice. TS-specific western blots were then performed with the immunoprecipitates. Purified rTS immunoprecipitated using rabbit α-TS served as the positive control. Serum obtained from both WT TS and TSKd1 null vaccinated mice pulled down both homologous and heterologous TS proteins, further supporting similar tertiary structures of wild type and TSKd1 null TS proteins. Deduced amino acid sequences of wild type TS and the TSKd1 null constructs were utilized to create structural 3D models using BioLuminate (Version 1.7, Schrödinger, LLC, New York, NY) and PyMOL (PyMOL Molecular Graphics System, Version 1.7.4 Schrödinger, LLC) as shown in (B). (TIF) [file ppat.1005896.s004.tif]

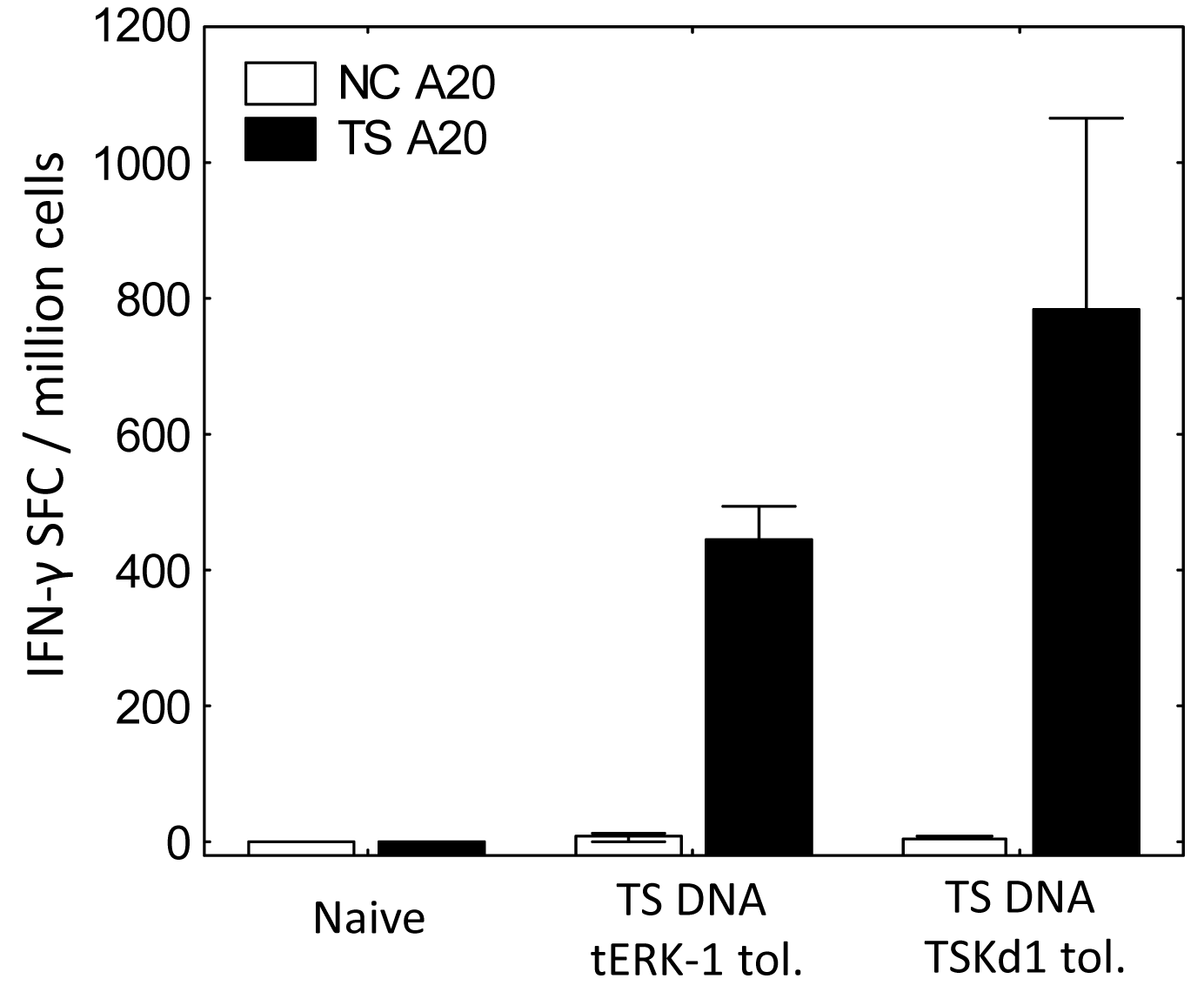

Supplement: S4 Fig — Large quantities of peptide (tERK-1 control and TSKd1, 100–300μg/dose) were injected i.v into BALB/c mice starting one week prior to wild type TS DNA vaccination (peptide i.v. on days -7, -3, -1, 7, 14, 21, 28, and 35 in relation to first TS DNA vaccination). Four weeks after the second and final immunization, spleen cells were removed and stimulated with APC (A20) transfected with the full length TS catalytic domain in overnight IFN-γ ELISPOT assays. (TIF) [file ppat.1005896.s005.tif]

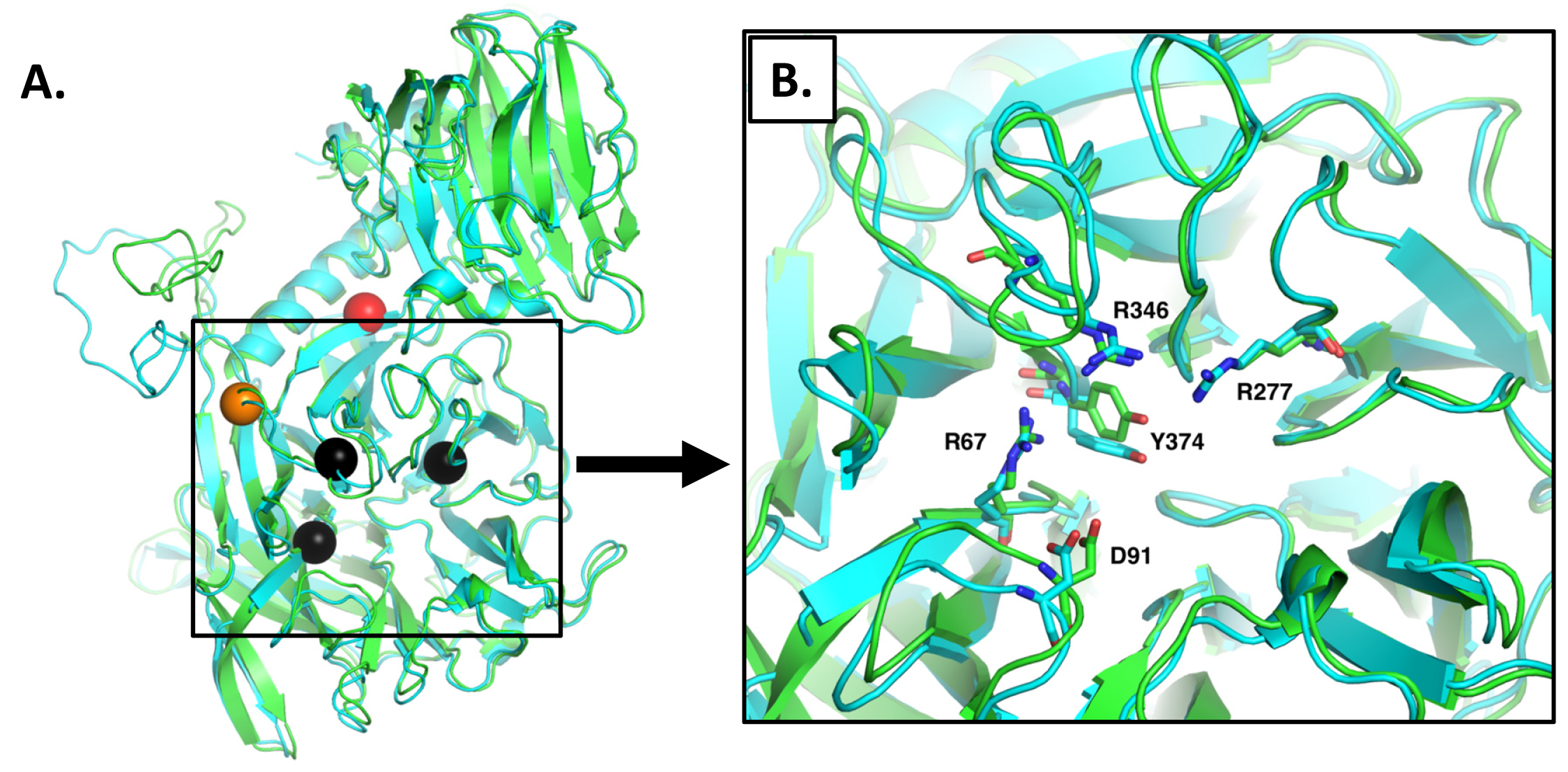

Supplement: S5 Fig — Structural models of wild type TS (green) and the TSKd1 null (magenta) constructs were created using BioLuminate and PyMOL. Highlighted in panel A are the 2 amino acids mutated to create the TSKd1 null vaccine (red and orange spheres represent the Y360G and I367F mutations, respectively). The black spheres depict the 3 arginine residues (R67, R277 and R346) which compromise the arginine triad important in binding TS substrates. In panel B, a merged enlarged view of the catalytic pocket predicted within WT TS and TSKd1 null TS is shown. Amino acid side chains of the 3 arginine residues (R67, R277, and R346) as well as 2 amino acids seemingly altered by mutation of TSKd1 (Y374 and D91) are noted. (TIF) [file ppat.1005896.s006.tif]

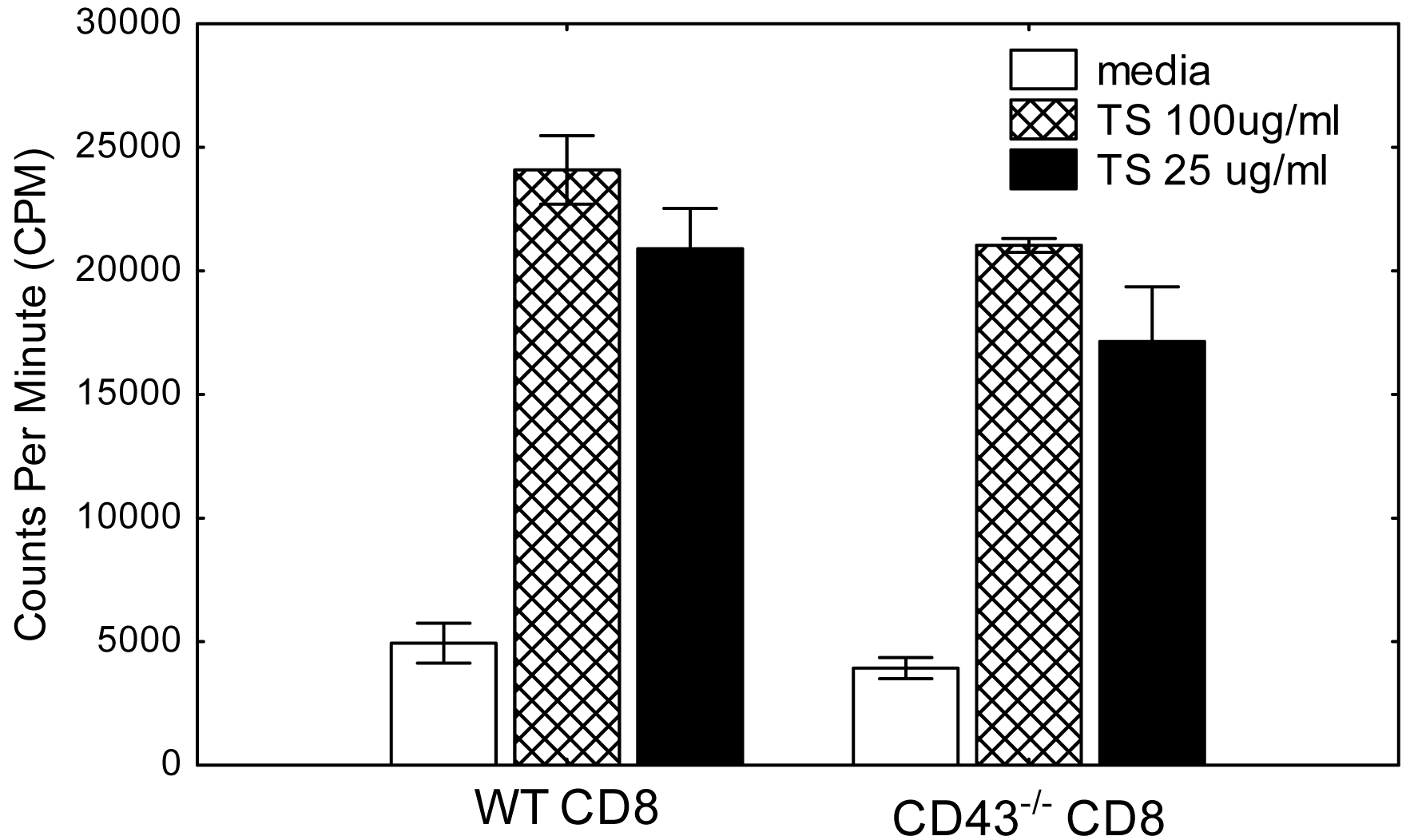

Supplement: S6 Fig — Naïve wild type (WT) B6 and naïve CD43-/- CD8+ T cells were purified by positive magnetic bead selection and incubated with suboptimal doses of PMA (12.5ng/ml) ± WT rTS (25–100μg/ml). After 3 days, proliferation was measured by 3H-Thymidine incorporation. (TIF) [file ppat.1005896.s007.tif]

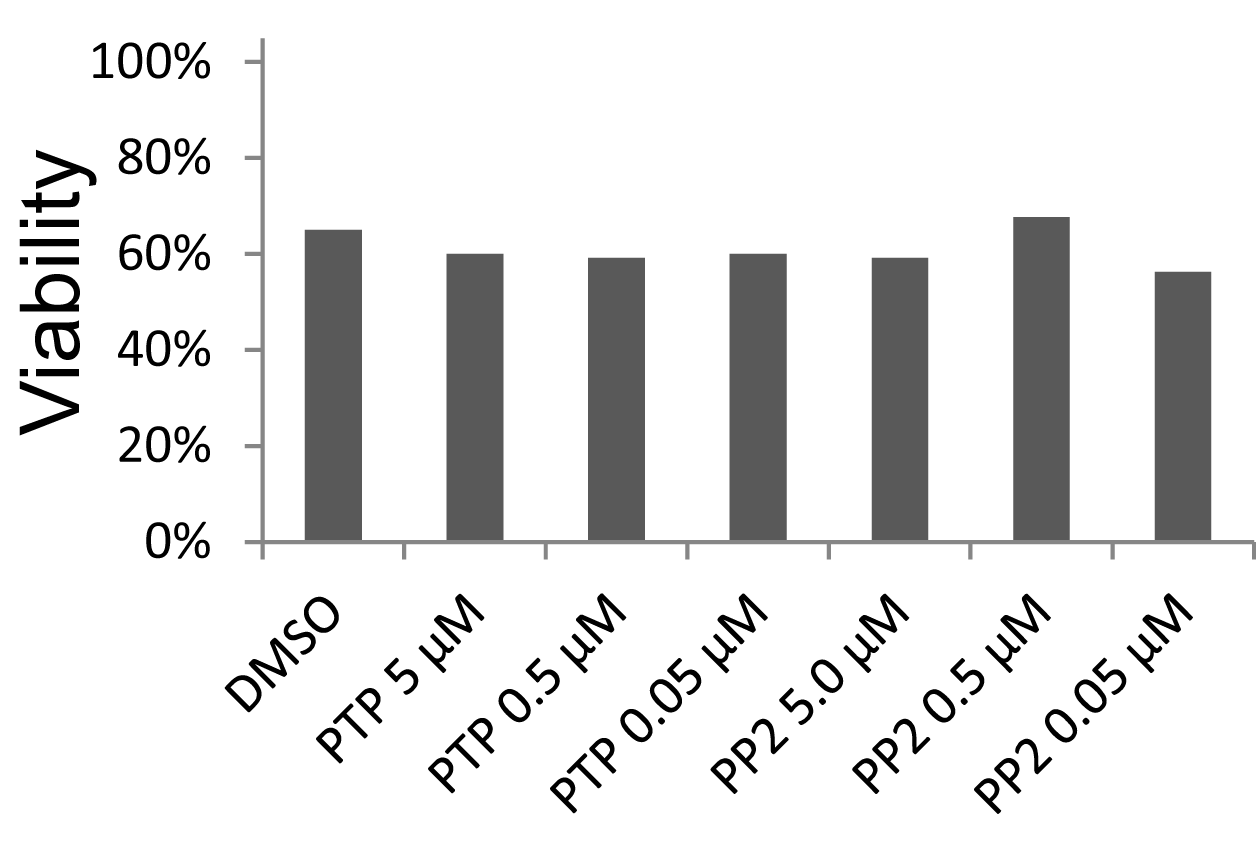

Supplement: S7 Fig — Naïve wild type BALB/c CD8+ T cells were purified by positive magnetic bead selection and incubated with suboptimal doses of PMA (12.5ng/ml) in the presence or absence of the indicated concentrations of CD45 inhibitor PTP or Src-family kinase inhibitor PP2. After 2 days of culture, cell viability was assessed by trypan blue exclusion microscopy. (TIF) [file ppat.1005896.s008.tif]
